# ﻿Seven new species of *Tetranemertes* Chernyshev, 1992 (Monostilifera, Hoplonemertea, Nemertea) from the Caribbean Sea, western Pacific, and Arabian Sea, and revision of the genus

**DOI:** 10.3897/zookeys.1181.109521

**Published:** 2023-10-05

**Authors:** Irina Cherneva, Christina I. Ellison, Eduardo E. Zattara, Jon L. Norenburg, Megan L. Schwartz, Juan Junoy, Svetlana A. Maslakova

**Affiliations:** 1 Department of Invertebrate Zoology, Faculty of Biology, Moscow State University, Moscow, Russia Moscow State University Moscow Russia; 2 Oregon Institute of Marine Biology and Biology Department, University of Oregon, Charleston, OR, USA University of Oregon Charleston United States of America; 3 Instituto de Investigaciones en Biodiversidad y Medio Ambiente, Centro Regional Universitario Bariloche, Universidad Nacional del Comahue, Consejo Nacional de Investigaciones Científicas y Tecnológicas, Bariloche, Argentina Universidad Nacional del Comahue Bariloche Argentina; 4 Department of Invertebrate Zoology, National Museum of Natural History, Smithsonian Institution, Washington, DC, USA Department of Invertebrate Zoology, National Museum of Natural History, Smithsonian Institution Washington United States of America; 5 School of Interdisciplinary Arts and Sciences, University of Washington, Tacoma, WA, USA University of Washington Tacoma United States of America; 6 Departamento de Ciencias de la Vida, Facultad de Ciencias, Universidad de Alcalá, Alcalá de Henares, Spain Universidad de Alcalá Alcala de Henares Spain

**Keywords:** Belize, description, histology-free, Nemertea, Oman, Panamá, revision, ribbon worm

## Abstract

The marine ribbon worm genus *Tetranemertes* Chernyshev, 1992 currently includes three species: the type species *T.antonina* (Quatrefages, 1846) from the Mediterranean Sea, *T.rubrolineata* (Kirsteuer, 1965) from Madagascar, and *T.hermaphroditica* (Gibson, 1982) from Australia. Seven new species are described: *T.bifrost***sp. nov.**, *T.ocelata***sp. nov.**, *T.majinbuui***sp. nov.**, and *T.pastafariensis***sp. nov.** from the Caribbean Sea (Panamá), and three species, *T.unistriata***sp. nov.**, *T.paulayi***sp. nov.**, and *T.arabica***sp. nov.**, from the Indo-West Pacific (Japan and Oman). As a result, an amended morphological diagnosis of the genus is offered. To improve nomenclatural stability, a neotype of *Tetranemertesantonina* is designated from the Mediterranean. The newly described species, each characterized by features of external appearance and stylet apparatus, as well as by DNA-barcodes, form a well-supported clade with *T.antonina* on a molecular phylogeny of monostiliferan hoplonemerteans based on partial sequences of COI, 16S rRNA, 18S rRNA, and 28S rRNA. Six of the seven newly described species, as well as *T.rubrolineata*, possess the unusual character of having a central stylet basis slightly bilobed to deeply forked posteriorly in fully grown individuals, a possible morphological synapomorphy of the genus. In addition, an undescribed species of *Tetranemertes* is reported from the Eastern Tropical Pacific (Panamá), increasing the total number of known species in the genus to eleven.

## ﻿Introduction

The monostiliferan hoplonemertean genus *Tetranemertes* is little-known even among the nemertean taxonomists, and currently includes only three valid species, two of which, *T.rubrolineata* (Kirsteuer, 1965) and *T.hermaphroditica* (Gibson, 1982), have not been seen since their original discovery in Madagascar and Australia, respectively. The third, and the type species of the genus, *T.antonina* (Quatrefages, 1846), has been recollected by one of us from the Mediterranean Sea (the type region), and DNA-barcoded by Kvist et al. (2014). However, the genus is apparently much more common and widespread in the world’s oceans, and this paper brings to light this little-known diversity and even wider geographic distribution.

The name *Tetranemertes* was proposed by Chernyshev (1992) as a replacement for *Nemertes* as defined by Friedrich (1955) and Kirsteuer (1974). The genus *Nemertes* was established by Cuvier (1817) for *Nemertesborlasii*, which was later synonymized with *Lineuslongissimus* (Gunnerus, 1770), a heteronemertean. Johnston (1837) and other authors used the name *Nemertes* for a group of monostiliferan hoplonemertean species, without designating a type species. The genus was redefined by Friedrich (1955), who included only the type species, *Nemertesantonina* Quatrefages, 1846. Verrill (1895) had assigned *N.antonina* to the genus *Emplectonema* Stimpson, 1857 but Friedrich (1955), in his revision of that genus, argued that *E.antonina* differs from other members of *Emplectonema* in body shape (thin and thread-like, rather than flattened and ribbon-like) and internal anatomy, and should be placed into a different genus. In particular, he noted that in *E.antonina* the longitudinal musculature of the body wall in the brain region comprises two layers (inner and outer) separated by a layer of parenchyma. He pointed out that this particular condition was only reported for one other species, the south African *Ommatopleaophiocephala* Schmarda, 1859 that was redescribed and placed into *Emplectonema* by Wheeler (1934), and synonymized the two despite considerable differences in external appearance and geographic distance (Friedrich 1955). Kirsteuer (1974) accepted Friedrich’s definition of *Nemertes*, and provided an updated diagnosis in his review of the monostiliferan genera with anteriorly split longitudinal musculature.

Chernyshev (1992) in a brief note on taxonomic names of several nemertean genera, pointed out that the type species of the genus *Nemertes* is not *Nemertesantonina* Quatrefages, 1846, but *Nemertesgracile* Johnston, 1837, and that *Nemertes* Friedrich, 1955 has two older homonyms (*Nemertes* Cuvier, 1817 and *Nemertes* Johnston, 1837). He argued that since *N.antonina* Quatrefages, 1846 is a valid taxon, the name *Nemertes* Friedrich, 1955 must be replaced, and proposed *Tetranemertes* as the replacement name for the genus whose type species is *N.antonina* Quatrefages, 1846.

The original description of *Tetranemertesantonina* is very brief, noting that the specimens are uniformly wine-red in color, have cephalic furrows, and four longitudinal rows of ocelli; Quatrefages (1846) apparently did not observe stylets. Bürger (1895) offered a much more detailed description of the species, including a color illustration of external appearance in life, as well as stylet apparatus. He noted the thin thread-like appearance of the body, unusually wide and short cerebral commissures of the brain, and small cerebral organs located far anterior to the brain. Bürger’s redescription and illustrations serve as the main reference for the identification of recently collected *T.antonina* specimens included in this study.

Here we describe four new species of *Tetranemertes* from the Caribbean Sea, one from the western Pacific Ocean (Japan) and Arabian Sea (Oman), and two from the Arabian Sea (Oman); we also report an additional species from the Eastern Tropical Pacific (Panamá), which at present cannot be formally described. The new species are placed into *Tetranemertes* based on morphological similarity to the other species of the genus, particularly to the type species *T.antonina* and to *T.rubrolineata*, the likes of which we recollected from the Arabian Sea for the first time since the original description. This placement is supported by a molecular phylogeny. We also re-evaluate the status of *Ommatopleaophiocephala* Schmarda, 1859 and designate a neotype of *T.antonina* from material recently collected in the Mediterranean Sea.

It has been the experience of senior co-authors, over decades of study, that most nemertean species can be unambiguously identified using a combination of characters of external appearance, stylet apparatus (in case of hoplonemerteans), reproductive characters, and DNA sequence data. At the same time, traditionally used characters of internal anatomy, reconstructed using time-consuming and artifact-prone histological techniques, are typically not helpful in distinguishing between closely related species. Others have similarly pointed this out and called for a change (e.g., Strand and Sundberg 2011; Strand et al. 2014; Sundberg 2015; Sundberg et al. 2016a, b), and this is now becoming an accepted practice in the field (e.g., Kajihara et al. 2018, 2022). This shift in taxonomic habits will expedite the rate of species descriptions, a much-needed change given hundreds of continuously discovered undescribed and cryptic species (e.g., Maslakova et al. 2022), and a rapidly changing world. Accordingly, our descriptions are intentionally focused on characters that are best assessed in life, e.g., shape, color, cephalic furrows, ocelli, stylet apparatus, backed by DNA-barcodes.

## ﻿Materials and methods

### ﻿Specimen collecting and preservation

Specimens were collected from the Mediterranean, Caribbean, the Gulf of Mexico, Eastern Tropical Pacific (Panamá), western Pacific (Japan), and the Arabian Sea (southern Oman) between 1999 and 2022. Recent collections (2018–2020) in Panamá were carried out under collecting permit numbers SE/A-55-18, SE/AP-9-2019, and specimens exported to Oregon Institute of Marine Biology, Oregon, USA (**OIMB**) for further study (export permit numbers SEX/A-20-2019, SEX/A-86-2019). Specimens from Oman were collected and exported to United States with permission from the Environment Authority of Oman (permit 6210/10/151). Characteristics of living specimens were documented on site with sketches and photographs, morphological vouchers were relaxed in 7.5% MgCl_2_, preserved in 10% buffered formalin for at least 24 h, post-fixed in Bouin’s for 48–72 h, then rinsed in tap water, and gradually transferred into 70% ethanol. Morphological vouchers for the newly described species, as well as tissue preserved for DNA barcoding are deposited at the National Museum of Natural History (**USNM**), Smithsonian Institution, Washington D.C., USA, in compliance with a Material Transfer Agreement with the Smithsonian Tropical Research Institute, Panamá. Specimens from Oman are deposited at the Florida Museum of Natural History (**FLMNH**). Tissue for molecular work was preserved in 95% ethanol and stored between -80 °C and -20 °C whenever possible. Specimen details, including tissue description, associated museum numbers, as well as GenBank Accession and BOLD IDs can be found in Table 1 and collecting information in Table 2, as well as in the published BOLD dataset (https://dx.doi.org/10.5883/DS-TETRA).

**Table 1.** Examined specimens of *Tetranemertes* species. USNM — National Museum of Natural History (Smithsonian Institution, USA), FLMNH — Florida Museum of Natural History (University of Florida, USA). OIMB — Oregon Institute of Marine Biology (University of Oregon, USA). MCZ-IZ — Museum of Comparative Zoology, Invertebrate Zoology (Harvard University, USA). MNCN — Museo National de Ciencias Naturales (Madrid, Spain). N/A – not available.

Species	Field ID	Collecting site	BOLD Process ID	Storing Institution and ID	Tissue description	GenBank Accession
***T.antonina* (Quatrefages, 1846)**	TE4_DR23	DR23	TETRW001-21	MCZ-IZ 132747	34 mm	CO1: KF935534 18S: KF935318 28S: KF935374 H3: KF935430
***T.antonina***	TE3_DR23	DR23	TETRW002-21	MNCN 5.02/28	5 mm specimen preserved in Bouine’s and stored in 100% ethanol	–
***T.arabica* sp. nov.**	BOMAN_08050	OM22-030B	NOMAN038-23	FLMNH 1087	anterior and posterior fixed for histology; midbody in 95% ethanol	CO1: OQ321721 18S: OQ322651 28S: OQ322596
	BOMAN_09099	OM22-051B	NOMAN039-23	OIMB	midbody in 95%, the rest of the specimen in RNA later	COI: OQ321713 18S: OQ322647 28S: OQ322593
	BOMAN_08029	OM22-022A	NOMAN040-23	FLMNH 1108	anterior and posterior fixed for histology; midbody in 95% ethanol	COI: OQ321711
	BOMAN_08030	OM22-022A	NOMAN041-23	FLMNH 1090	anterior and posterior fixed for histology; midbody in 95% ethanol	COI: OQ321712
	BOMAN_08031	OM22-022A	NOMAN042-23	FLMNH 1084	anterior and posterior fixed for histology; midbody in 95% ethanol	COI: OQ321716
	BOMAN_08074	OM22-022A	NOMAN043-23	FLMNH 1113	anterior and posterior fixed for histology; midbody in 95% ethanol	COI: OQ321717
	BOMAN_08075	OM22-022A	NOMAN044-23	FLMNH 1115	anterior and posterior fixed for histology; midbody in 95% ethanol	COI: OQ321710
	BOMAN_08300	OM22-031B	NOMAN045-23	FLMNH 1085	anterior and posterior fixed for histology; midbody in 95% ethanol	COI: OQ321709
***T.bifrost* sp. nov.**	852_080203_1	BdT03	CARNE027-19	N/A	N/A	CO1: MW021890 16S: MW022069
	852_080203_2	BdT03	CARNE028-19	OIMB	fragment of body in 95% ethanol	16S: MW022070
	852_080203_3	BdT03	CARNE029-19	OIMB	fragment of body in 95% ethanol	CO1: MW021891 16S: MW022071
	852_080203_4	BdT03	CARNE030-19	OIMB	fragment of body in 95% ethanol	CO1: MW021892 16S: MW022072
	852_080203_8	BdT03	CARNE031-19	USNM 1156475	histological sections of head and midbody; posterior in 95% ethanol	16S: MW022073
	CB057_18_01	B2018-21	CARNE498-21	USNM 1618745	whole body in 95% ethanol	CO1: MT578867 16S: MT581161 18S: MT581203 28S: MT581189
	CB057_18_03	B2018-21	CARNE499-21	USNM 1618747	whole body in 95% ethanol	CO1: MT578868 16S: MT581162
	CB057_18_04	B2018-21	CARNE500-21	USNM 1618748	whole body in 95% ethanol	CO1: MT578869 16S: MT581163
	CB057_18_05	B2018-03B	CARNE501-21	N/A	N/A	CO1: MT578870 16S: MT581164
	CB057_18_06	B2018-21	CARNE502-21	N/A	N/A	CO1: MT578871 16S: MT581165
	CB057_18_07	B2018-21	CARNE503-21	USNM 1618678	anterior fixed for histology; posterior in 95% ethanol	CO1: MT578872 16S: MT581166 18S: MT581204 28S: MT581190
	CBdT0062	BdT19-09	CARNE504-21	USNM 1660660	anterior fixed for histology; posterior in 95% ethanol	CO1: MT578873
	CBdT0063	BdT19-08	CARNE505-21	USNM 1660661	anterior fixed for histology; posterior in 95% ethanol	CO1: MT578874 16S: MT581167
	CBdT0064	BdT19-08	CARNE506-21	USNM 1660662	anterior fixed for histology; posterior in 95% ethanol	CO1: MT578875 16S: MT581168
	852_080203_5	BdT03	TETRW003-21	USNM 1156472	histological sections	–
***T.bifrost* sp. nov.**	852_080203_6	BdT03	TETRW004-21	USNM 1156473	histological sections	–
	852_080203_7	BdT03	TETRW005-21	USNM 1156474	histological sections	–
	852_080203_9	BdT03	TETRW006-21	USNM 1156476	histological sections	–
	CB057_18_02	B2018-21	TETRW007-21	USNM 1618746	whole body in 95% ethanol	–
	SMCP0366	BdT19-25	TETRW017-21	USNM 1660664	anterior and midbody fixed for histology, and posterior in 95% ethanol	–
	SMCP0397	BdT19-25	TETRW018-21	USNM 1660666	anterior and midbody fixed for histology, and posterior in 95% ethanol	–
	SMCP1970	BdT19-19	TETRW025-21	USNM 1660837	whole body in 95% ethanol	–
***T.majinbuui* sp. nov.**	1502	PR	CARNE025-19	N/A	N/A	CO1:MW021889
	856_080303_3	BdT03R	CARNE026-19	USNM 1156478	histological slides: frontal sections of anterior	16S: MW022068
	CB056_18_01	B2018-04	CARNE495-21	USNM 1618677	anterior fixed for histology; posterior in 95% ethanol	CO1: MT578864 16S: MT581160 18S: MT581201 28S: MT581187
	CB056_18_02	B2018-04	CARNE496-21	USNM 1618744	whole body in 95% ethanol	CO1: MT578865 18S: MT581202 28S: MT581188
	CBdT0058	BdT19-09	CARNE497-21	USNM 1660658	anterior fixed for histology; posterior in 95% ethanol	CO1: MT578866
	856_080303_1	BdT03	TETRW008-21	USNM 1156477	histological sections	–
	856_080303_4	BdT03	TETRW010-21	USNM 1156479	histological sections	–
	SMCP0372	BdT19-25	TETRW016-21	USNM 1660665	anterior fixed for histology, posterior in 95% ethanol	–
	SMCP1402	BdT19-20	TETRW019-21	USNM 1660831	whole body in 95% ethanol	–
	SMCP1407	BdT19-21	TETRW020-21	USNM 1660832	whole body in 95% ethanol	–
	SMCP1438	BdT19-20	TETRW021-21	USNM 1660833	whole body in 95% ethanol	–
	SMCP1439	BdT19-20	TETRW022-21	USNM 1660834	whole body in 95% ethanol	–
	SMCP1440	BdT19-20	TETRW023-21	USNM 1660835	whole body in 95% ethanol	–
	SMCP1952	BdT19-21	TETRW024-21	USNM 1660836	whole body in 95% ethanol	–
	SMCP1973	BdT19-21	TETRW026-21	USNM 1660838	whole body in 95% ethanol	–
	SMCP1974	BdT19-21	TETRW027-21	USNM 1660839	whole body in 95% ethanol	–
	SMCP1992	BdT19-20	TETRW028-21	USNM 1660840	whole body in 95% ethanol	–
***T.ocelata* sp. nov.**	685_061202_1	CBC02	CARNE022-19	USNM 1156294	histological sections	CO1: MW021887 16S: MW022065
	685_061202_2	CBC02	CARNE023-19	USNM 1156296	transverse histological sections of anterior	CO1: MW021888 16S: MW022066
	685_061202_3	CBC02	CARNE024-19	USNM 1156297	histological sections; tissue in 95% ethanol	16S: MW022067
	685_061202_4	CBC02	TETRW011-21	USNM 1156298	histological sections	–
	995_070906_01	GM06	TETRW012-21	USNM 1112806	anterior in formalin, posterior in RNAlater	CO1: MT578863 16S: MT581159 18S: MT581200 28S: MT581186
***T.pastafariensis* sp. nov.**	CB055_18_01	B2018-03B	CARNE524-21	USNM 1618739	whole body in 95% ethanol	CO1: MT578887 16S: MT581177 18S: MT581207 28S: MT581193
	CB055_18_02	B2018-03B	CARNE525-21	USNM 1618740	whole body in 95% ethanol	CO1: MT578888 16S: MT581178 18S: MT581208 28S: MT581194
	CB055_18_03	B2018-03B	CARNE526-21	USNM 1618741	whole body in 95% ethanol	CO1: MT578889 16S: MT581179
	CB055_18_04	B2018-03B	CARNE527-21	USNM 1618675	anterior fixed for histology; posterior in 95% ethanol	CO1: MT578890 16S: MT581180 18S: MT581209 28S: MT581195
	CB055_18_05	B2018-03B	CARNE528-21	USNM 1618750	posterior in 95% ethanol	CO1: MT578891 16S: MT581181
***T.pastafariensis* sp. nov.**	CB055_18_06	B2018-20	CARNE529-21	USNM 1618676	anterior fixed for histology, and posterior in 95% ethanol	CO1: MT578892 18S: MT581210 28S: MT581196
	CB055_18_07	B2018-20	CARNE530-21	USNM 1618742	whole body in 95% ethanol	CO1: MT578893
	CB055_18_08	B2018-20	CARNE531-21	USNM 1618743	whole body in 95% ethanol	CO1: MT578894 16S: OK073428
	SMCP0019	BdT19-13	CARNE532-21	USNM 1660663	anterior fixed for histology; posterior in 95% ethanol	CO1: MT578896 16S: MT581182
	CBdT0059	BdT19-09	CARNE573-21	USNM 1660659	anterior fixed for histology; posterior in 95% ethanol	CO1: MT578895
***T.paulayi* sp. nov.**	BOMAN_07013	OM22-011A	NOMAN049-23	FLMNH 1055	anterior and posterior fixed for histology; midbody in 95% ethanol	COI: OQ321715 18S: OQ322649
	BOMAN_08291	OM22-031B	NOMAN050-23	FLMNH 1118	anterior and posterior fixed for histology; midbody in 95% ethanol	18S: OQ322646 28S: OQ322592
	BOMAN_08284	OM22-031B	NOMAN051-23	FLMNH 1141	anterior fixed for histology, posterior in 95% ethanol	COI: OQ321720
	BOMAN_08302	OM22-031B	NOMAN052-23	FLMNH 1082	anterior fixed for histology, posterior in 95% ethanol	COI: OQ321722
	BOMAN-08289	OM22-031C	–	FLMNH 1136	anterior and posterior fixed for histology; midbody in 95% ethanol	–
	BOMAN-08301	OM22-31B	–	FLMNH 1133	anterior and posterior fixed for histology; midbody in 95% ethanol	–
	BOMAN-09064	OM22-37B	–	OIMB	whole body in 95% ethanol, bulk	–
	BOMAN-09065	OM22-37B	–	OIMB	whole body in 95% ethanol, bulk	–
	BOMAN-09066	OM22-37B	–	OIMB	whole body in 95% ethanol, bulk	–
	BOMAN-09067	OM22-37B	–	OIMB	whole body in 95% ethanol, bulk	–
***T.rubrolineata* (Kirsteuer, 1965)**	BOMAN_08053	OM22-030B	NOMAN046-23	FLMNH 1089	anterior fixed for histology, posterior in 95% ethanol	COI: OQ321714
	BOMAN_08078	OM22-022A	NOMAN047-23	FLMNH 1126	anterior and posterior fixed for histology	COI: OQ321723
	BOMAN_08038	OM22-022A	NOMAN048-23	FLMNH 1094	anterior and posterior fixed for histology; midbody in 95% ethanol	COI: OQ321718 18S: OQ322650 28S: OQ322595
***T.* sp. ETP001**	SMPP0632	PP20	NOPP001-21	N/A	N/A	CO1: MT578897 16S: MT581183 18S: MT581211 28S: MT581197
***T.unistriata* sp. nov.**	BOMAN_07032	OM22-011A	NOMAN037-23	FLMNH 1062	anterior and posterior fixed for histology	COI: OQ321719
	490_070999_1	JP99	TETRW013-21	USNM 1011573	histological slides: cross sections of posterior; tissue in 95% ethanol	CO1: MT578861 16S: MT581158 18S: MT581198 28S: MT581184
	490_071099_2	JP99	TETRW014-21	USNM 1011574	histological slides: longitudinal sections of posterior; tissue in 95% ethanol	CO1: MT578862 18S: MT581199 28S: MT581185
	490_071099_3	JP99	TETRW015-21	USNM 1011575	histological sections of head and posterior	–

**Table 2.** Collecting information for *Tetranemertes* specimens.

Site	Date	Location	GPS	Habitat	Depth	Collectors
**B2018-03B**	21 Aug 2018	Hospital Point, Isla Solarte, Bocas del Toro, Panamá	9.333755, -82.218583	coral rubble	1–2 m	Megan Schwartz et al.
**B2018-04**	22 Aug 2018	Wild Cane Key, Isla Bastimentos, Bocas del Toro, Panamá	9.348691, -82.168114	coral rubble	5–7 m	Svetlana Maslakova, Maycol Madrid
**B2018-20**	28 Aug 2018	Hospital Point, Isla Solarte, Bocas del Toro, Panamá	9.333755, -82.218583	coral rubble	1–2 m	Megan Schwartz et al.
**B2018-21**	22 Aug 2018	Mangrove Point, Isla Colon, Bocas del Toro, Panamá	9.327247, -82.253636	coral rubble	1–2 m	Irina Cherneva, Eduardo Zattara, Christina Ellison
**BdT03**	2-3 Aug 2003	Bocas del Toro, Panamá	N/A	N/A	N/A	Jon Norenburg
**BdT03R**	3 Aug 2003	Cayo Roldan, Bocas del Toro, Panamá	N/A	N/A	N/A	Jon Norenburg
**BdT19-08**	8 Aug 2019	Playa Escondida 2, Isla Colón, Bocas del Toro, Panamá	9.379778, -82.238778	sandy-muddy beach with rocks	1–2 m	Christina Ellison, Maycol Madrid
**BdT19-09**	9 Aug 2019	Playa Boca del Drago, Isla Colón, Bocas del Toro, Panamá	9.414639, -82.331167	sandy beach with rocks, rubble	1–2 m	Christina Ellison, Maycol Madrid
**BdT19-13**	26 Aug 2019	Cayo Coral, Isla Bastimentos, Bocas del Toro, Panamá	9.243583, -82.111972	very lively reef, with lots of green calcareous (and other types of) algae	7–8 m	Svetlana Maslakova, Deyvis González, Christina Ellison
**BdT19-19**	3 Sep 2019	Fuerte Sherman, Colón, Panamá	9.3532872, -79.9449529	fossil reef, silted coral rubble, mostly solid	3–4 m	Svetlana Maslakova, Maycol Madrid
**BdT19-20**	4 Sep 2019	Drake Island, Portobelo, Colón, Panamá	9.561389, -79.685	Live reef with lots of Agaricia rubble	12 m	Svetlana Maslakova, Maycol Madrid
**BdT19-21**	4 Sep 2019	Huerta, Portobelo, Colón, Panamá	9.561944, -79.681389	Live reef with lots of Agaricia rubble	8–15 m	Svetlana Maslakova, Maycol Madrid
**BdT19-25**	10 Sep 2019	Playa Boca del Drago, Isla Colón, Bocas del Toro, Panamá	9.414639, -82.331167	Sandy beach, intertidal/shallow subtidal – rocks, rubble	1–2 m	Christina Ellison, Maycol Madrid
**CBC02**	12 Jun 2002	Carrie Bow Cay, Belize	N/A	coral rubble with orange sponge	30 m	Megan Schwartz Jon Norenburg
**DR23**	25 Sep 2011	Alboran Island, Spain	35.932167, -3.040333	coralline red algae and *Ulva*	25 m	Juan Junoy
**GM06**	26 Jul 2006	NSF III Sta. 89, Northern Gulf of Mexico	27.9850, -91.6295	shell riddled with sponge (collected with box dredge)	65–71 m	Jon Norenburg
**JP99**	9-10 Jul 1999	Engetsu Island, Shirahama, Wakayama, Japan	33.689, 135.336	among calcareous algae	1–2 m	Svetlana Maslakova, Megan Schwartz
**OM22-011A**	11 Jan 2022	Hamdis, Mirbat, Dhofar, Oman	16.94735, 54.76255	coral rubble	4–9 m	Svetlana Maslakova
**OM22-022A**	16 Jan 2022	Hamdis, Mirbat, Dhofar, Oman	16.94735, 54.76256	coral rubble	6 m	Svetlana Maslakova, Gustav Paulay
**OM22-030B**	19 Jan 2022	Michel’s Reef, Mirbat, Dhofar, Oman	16.94332, 54.73005	shell hash	30 m	Svetlana Maslakova
**OM22-031B**	20 Jan 2022	Inshore of Chinese Wreck, Mirbat, Dhofar, Oman	16.96612, 54.70797	scraped sample of vermetid/coralline algae encrustation	2 m	Svetlana Maslakova, Gustav Paulay
**OM22-031C**	20 Jan 2022	Inshore of Chinese Wreck, Mirbat, Dhofar, Oman	16.96612, 54.70798	algal holdfasts	2 m	Svetlana Maslakova, Gustav Paulay
**OM22-037B**	22 Jan 2022	Roshan Reef, Mirbat, Dhofar, Oman	16.96852, 54.69022	from barnacle and algae scraping	7–8 m	Gustav Paulay
**OM22-051B**	26 Jan 2022	Chinese Wreck, Mirbat, Dhofar, Oman	16.96612, 54.70797	coral rubble	3–5 m	Svetlana Maslakova, Gustav Paulay
**PP20**	20 Jan 2020	Isla Pachequilla, Panamá, Panamá	8.67231, -79.0605	coral rubble	5 m	Christina Ellison, Maycol Madrid
**PR**	Aug 2011	Vieques, Puerto Rico	N/A	N/A	0–2 m	Megan Schwartz

### ﻿DNA extraction and PCR

Total genomic DNA was extracted using DNEasy Blood and Tissue Kit (Qiagen). DNA extracts are kept in the Maslakova lab at the Oregon Institute of Marine Biology. Partial sequences of two mitochondrial genes, Cytochrome Oxidase I (COI) and 16S rDNA, and two nuclear genes, 18S rDNA and 28S rDNA, were PCR-amplified using primers listed in Table 3. Polymerase chain reactions were performed in 20-μl volumes, with 1 unit per reaction of Go Taq Polymerase (Promega) with supplied buffer, 200 μM dNTPs, and 500 nM of each primer. Thermal cycling was initiated with 2 min at 95 °C, followed by 34 cycles of 40 s at 95 °C, annealing at 43–45 °C (COI), 45–50 °C (16S), 55 °C (18S), 55 °C (28S) and extension at 72 °C for 1 min. Reaction was terminated with a 2-min final elongation at 72 °C. PCR products were purified using SV Wizard Gel and PCR Clean up Kit (Promega), and sent to Sequeteq, Inc. (Mountain View, CA, USA) to be sequenced in both directions using PCR primers. Sequences were trimmed to eliminate primers and low-quality regions, and overlapping fragments assembled into contigs using Geneious 11.0.4 (Biomatters). Consensus sequences were proofread based on quality scores and any bases with cumulative quality Phred scores of less than 20 (i.e., probability of erroneous base call > 0.01) were converted to “N”s. Cytochrome Oxidase I sequences were translated using Invertebrate Mitochondrial translation table, and checked for stop codons. Sequence identity was verified using nucleotide BLAST tool (NCBI), and any non-nemertean sequences eliminated as contamination. DNA sequences are deposited in BOLD and GenBank (see Table 1, BOLD dataset https://dx.doi.org/10.5883/DS-TETRA).

**Table 3.** PCR primers used in this study.

Gene	Primer	Sequence	Annealing T	Source
**COI**	LCO1490	5’ GGTCAACAAATCATAAAGATATTGG	43–45 °C	Folmer et al. 1994
	HCO2198	5’ TAAACTTCAGGGTGACCAAAAAATCA		Folmer et al. 1994
**COI**	CO1LF	5’ TTTCAACAAATCATAAAGATAT	43–45 °C	Norenburg, unpublished
	CO1DR	5’ GAGAAATAATACCAAAACCAGG		Norenburg, unpublished
**16S rRNA**	16SARL	5’ CGCCTGTTTATCAAAAACAT	45–50 °C	Palumbi et al. 1991
	16SBRH	5’ CCGGTCTGAACTCAGATCACGT		Palumbi et al. 1991
**16S rRNA**	16SAF	5’ TCGTCTGTTTATCAAAAACATAGY	45–50 °C	Norenburg unpublished
	16SKR	5’ AATAGATAGAAACCAACCTGGC		Norenburg unpublished
**18S rRNA**	18SF1	5’ CTGGTGCCAGCAGCCGCGGYAA	55 °C	Machida and Knowlton 2012
	18SRC2	5’ TCCGTCAATTYCTTTAAGTT		Machida and Knowlton 2012
**28S rRNA**	LSU3	5’ TCCTGAGGGAAACTTCGG	55 °C	Littlewood 1994
	LSU5	5’ ACCCGCTGAAYTTAAGCA		Littlewood 1994

### ﻿Sequence alignment, phylogenetic analyses, and barcoding gap analysis

COI, 16S, 18S, and 28S sequences for the palaeonemerteans *Cephalothrixbipunctata* and *Carinoma* sp. and for the hoplonemerteans *Paradrepanophoruscrassus*, *Prostomaeilhardi*, *Nemertopsisbivittata*, *Zygonemertesalbida*, *Ototyphlonemertespallida*, *Potamonemertespercivali*, *Antarctonemertesriesgoae*, *Vieitezialuzmurubeae*, *Oerstedia* sp., and *Tetranemertesantonina* were downloaded from GenBank (see Table 4 for accession numbers). A multiple sequence alignment (MSA) for each marker was initially estimated automatically using MAFFT v. 7.017 (Katoh and Standley 2013) with default settings; each MSA was inspected and curated by eye, and then the MSAs from the four markers were concatenated. The MSA was analyzed with RAxML v. 8.2.11 (Stamatakis 2014), set up to perform 1000 rapid bootstrap inferences followed by a thorough maximum likelihood search, using a General Time Reversible (GTR) model with gamma-distributed rate heterogeneity. The MSA was divided into six partitions, each run with different models: three partitions for the protein coding marker COI (one partition for each codon position) and one partition for each of the rRNA markers (16S, 18S, and 28S). Bayesian inference from the MSA was also performed using MrBayes 3.2.6 (Ronquist et al. 2012), specifying a GTR model with 4 categories of gamma distributed rate heterogeneity and a proportion of invariant sites. Four heated chains were run for 1,100,000 steps and subsampled every 200 steps; the initial 100,000 steps were discarded as burn-in. COI sequences from 49 specimens (including all new specimens in the previous analysis plus additional specimens), along with accessions KF935534 (*Tetranemertesantonina*) and KF935533 (*Tetranemertesbifrost* sp. nov.), were aligned using MAFFT into a 627 bp MSA. The MSA was used as input for Automatic Barcode Gap Discovery (ABDG) (Puillandre et al. 2012). ABDG was run with default options. Pairwise Jukes-Cantor distances were calculated using the *dist.dna* function from the *ape* package (Paradis and Schliep 2019) in R (R Core Team 2020).

**Table 4.** Previously published sequences used in phylogenetic analysis.

Species	MCZ-IZ#	CO1	16S	18S	28S	Source
***Cephalothrixbipunctata***	133009	KF935501	KF935447	KF935279	KF935335	Kvist et al. 2014
***Carinoma* sp.**	135341	KF935500	KF935446	KF935278	KF935334	Kvist et al. 2014
***Antarctonemertesriesgoae* Taboada et al., 2013**	134229	KF935538	KF935490	KF935322	KF935378	Kvist et al. 2014
***Nemertopsisbivittata***		MK047680	MK067304	MK076305	MK076426	Zattara et al. 2019
***Oerstedia* sp.**	132740	KF935535	KF935487	KF935319	KF935375	Kvist et al. 2014
***Ototyphlonemertespallida***	133745	KF935545	KF935496	KF935329	KF935385	Kvist et al. 2014
***Paradrepanophoruscrassus***	DNA104800	HQ848603	JF277628	JF293008	HQ856867	Andrade et al. 2012
***Potamonemertespercivali***	25172	KF935532	KF935483	KF935316	KF935372	Kvist et al. 2014
***Prostomaeilhardi* (Montgomery, 1894)**	DNA103928	HQ848594	JF277620	JF293027	HQ856875	Andrade et al. 2011
***Tetranemertesantonina***	132747	KF935534		KF935318	KF935374	Kvist et al. 2014
***Tetranemertesbifrost* sp. nov.**	133023	KF935533	KF935484	KF935317	KF935373	Kvist et al. 2014
***Vieitezialuzmurubeae* Junoy, Andrade & Giribet, 2010**	133740	KF935544	KF935495	KF935328	KF935384	Kvist et al. 2014
***Zygonemertesalbida* Coe, 1901**		MK047684	MK067308	MK076309	MK076430	Zattara et al. 2019

## ﻿Results

### ﻿Phylogenetic analysis

**Figure 1.**
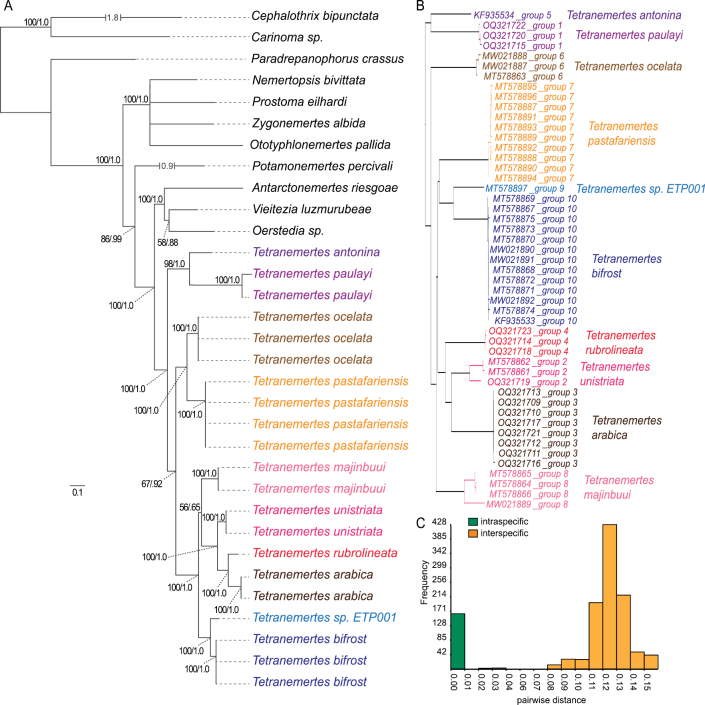
Phylogeny and species delimitation of *Tetranemertes***A** maximum Likelihood phylogeny based on concatenated dataset of Cytochrome Oxidase I, 16S rRNA, 18S rRNA and 28S rRNA sequences. Clade support is shown as likelihood as well as posterior probability (from Bayesian analysis) **B**ABGD grouping tree **C** histogram of pairwise distances calculated from partial Cytochrome Oxidase I multiple alignment, showing intraspecific and interspecific pairs.

Alignment of four phylogenetic markers from a total of 32 specimens (13 from GenBank and 19 newly sequenced) yielded four multiple sequence alignments (MSAs; COI: 709 bp; 16S: 563 bp; 18S: 1820 bp; 28S: 3306 bp), which were concatenated into a single MSA 6380 bp long. The MSA was used as input for maximum likelihood (ML) and Bayesian (BI) phylogenetic inference. The resulting ML and BI trees are mostly congruent and well supported (Fig. 1A); the subtree containing all species of *Tetranemertes* is topologically identical for both inference methods, recovering a monophyletic *Tetranemertes* with 100% bootstrap support and 1.0 posterior probability. Furthermore, all putative species for which we had more than a single sequence were found to be reciprocally monophyletic with full support (100% bootstrap/1.0 posterior probability).

### ﻿Species delimitation

To test if patterns of genetic divergence agreed with morphologically defined species, additional COI sequences were obtained from *Tetranemertespastafariensis* sp. nov., *T.majinbuui* sp. nov., *T.bifrost* sp. nov., *T.arabica* sp. nov., *T.unistriata* sp. nov., *T.rubrolineata* sp. nov., and *T.paulayi* sp. nov. individuals and added to the existing COIMSA. After removing all non-*Tetranemertes* sequences and trimming off low coverage ends, the resulting 49-sequence, 627-bp length MSA was used as input for an automated barcoding gap analysis (ABGD). ABDG analyses with and without recursion strongly supported a total of ten different groups, each of them matching morphological species hypotheses (Fig. 1B). When categorizing all pairwise Jukes-Cantor distances as intra- or interspecific based on our morphological species assignments, all intraspecific distances were below 0.04 while all interspecific distances were above 0.08 (Fig. 1C; Table 5).

**Table 5.** Averaged Jukes-Cantor distances for all possible pairwise intraspecific (diagonal, bold) and interspecific (above diagonal) comparisons of Cytochrome Oxidase I sequences of *Tetranemertes* species. Assignments based on morphological species hypotheses. The asterisk (*) marks species represented by sequence data for only one individual.

	*T.antonina*	*T.arabica*	*T.bifrost*	*T.majinbuui*	*T.ocelata*	*T.pastafariensis*	*T.paulayi*	*T.rubrolineata*	*T.* ETP001	*T.unistriata*
*T.antonina*	*	0.148	0.146	0.127	0.127	0.142	0.114	0.138	0.135	0.148
*T.arabica*		**0.000**	0.133	0.110	0.113	0.120	0.129	0.112	0.101	0.098
*T.bifrost*			**0.001**	0.118	0.129	0.128	0.146	0.123	0.077	0.113
*T.majinbuui*				**0.015**	0.122	0.129	0.128	0.136	0.103	0.126
*T.ocelata*					**0.002**	0.119	0.132	0.117	0.123	0.115
*T.pastafariensis*						**0.001**	0.159	0.123	0.127	0.131
*T.paulayi*							**0.002**	0.143	0.120	0.163
*T.rubrolineata*								**0.000**	0.130	0.103
*T.* sp. ETP001									*	0.125
*T.unistriata*										**0.015**

### ﻿Systematics

**Class Hoplonemertea Hubrecht, 1879**

**Order Monostilifera Brinkmann, 1917**

**Family Oerstediidae Chernyshev, 1993**

Kajihara (2021) in his recent revision of monostiliferan families placed *Tetranemertes* within the Oerstediidae based on the phylogenetic analysis by Kvist et al. (2014).

#### ﻿Genus Tetranemertes

Taxon classificationAnimaliaMonostiliferaOerstediidae

Chernyshev, 1992

9D5E1839-F18C-509D-A327-0C683E4B25BE

##### Type species.

*Tetranemertesantonina* (Quatrefages, 1846), by monotypy.

##### Diagnosis.

Body long and thin, thread-like; head not demarcated from body; numerous ocelli arranged in four longitudinal rows (two on each side of head, the two rows almost on top of one another); cerebral organs small, located far in front of brain; a pair of shallow oblique or transverse cerebral organ furrows restricted to ventral and lateral surfaces, fused mid-ventrally forming a single furrow; cerebral commissures unusually short and wide; lateral nerve cords with a single fibrous core; longitudinal musculature anteriorly divided by a layer of connective tissue, only the inner layer contributes to proboscis insertion, i.e. precerebral septum lacking; rhynchocoel between 1/5 and 1/3 of body length; proboscis short and thin, with a neural sheath rather than distinct proboscis nerves; anterior proboscis very short, stylets located very close to head, often within a few millimeters of cerebral ganglia. The seven new species described below, as well as *Tetranemertesrubrolineata*, possess an unusual character of having the central stylet’s basis posteriorly slightly bilobed to deeply forked in fully grown individuals.

##### Composition.

The genus includes ten described species: *Tetranemertesantonina* (Quatrefages, 1846), *T.rubrolineata* (Kirsteuer, 1965), *T.hermaphroditica* (Gibson, 1982), *T.bifrost* sp. nov., *T.majinbuui* sp. nov., *T.pastafariensis* sp. nov., *T.unistriata* sp. nov., *T.ocelata* sp. nov., *T.paulayi* sp. nov., *T.arabica* sp. nov., and one undescribed species (*Tetranemertes* sp. ETP001). *Ommatopleaophiocephala* Schmarda, 1859 from South Africa, previously synonymized with *T.antonina* by Friedrich (1955), is almost certainly a distinct species, most likely not related to *Tetranemertes* (see Discussion).

##### Geographic distribution.

Mediterranean Sea (Banyuls, Trieste, Sicily, Naples, Alborán Island, Almería, Strait of Gibraltar), Caribbean Sea (Bocas del Toro, Panamá; Carrie Bow Cay, Belize; Puerto Rico, USA), western Indian Ocean (Madagascar), Arabian Sea (Dhofar Governorate, Oman), Western Pacific (Heron Island, Australia and Honshu Island, Japan), Eastern Tropical Pacific (Panamá).

##### Etymology.

The name refers to the number of times the genus *Nemertes* was re-defined: *Nemertes* Cuvier, 1817, *Nemertes* Johnston, 1837, *Nemertes* Friedrich, 1955, and *Nemertes* Kirsteuer, 1974.

#### ﻿ Tetranemertes antonina

Taxon classificationAnimaliaMonostiliferaOerstediidae

(Quatrefages, 1846)

EFF5A9CA-2C39-5DD0-A28F-C538C26FC363

Fig. 2

Nemertes antonina Quatrefages, 1846: 111; Diesing 1850: 274; Hubrecht 1879: 231; Joubin 1890: 590; Friedrich 1955: 171; Kirsteuer 1965: 315; Gibson 1982: 285.
Eunemertes antonina : Joubin 1894: 206; Bürger 1895: 38.
Emplectonema antonina : Friedrich 1955: 171.
Polia antonina : Hubrecht 1879: 231.

##### Material examined.

**Figure 2.**
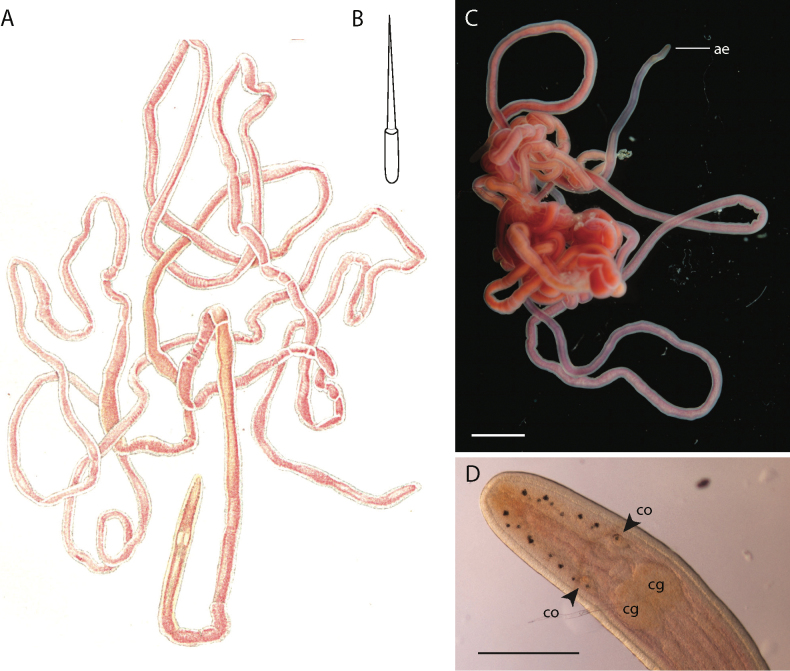
*Tetranemertesantonina***A** external appearance of *T.antonina*, after Bürger (1895)**B** central stylet of *T.antonina* after Bürger (1895). *Tetranemertesantonina* neotype (specimen: TR3-DR23, Museum ID: MNCN 5.02/28) **C** external appearance of living specimen and **D** head in transmitted light showing distribution and number of ocelli. Abbreviations: ae — anterior end, cg — cerebral ganglion, co — cerebral organ. Scale bars: 4 mm (**C**); 0.75 mm (**D**).

Type material was never designated, and it is very unlikely that either Quatrefages’ or Bürger’s specimens exist or can be found. With the purpose of improving nomenclatural stability, we designate the following specimen examined by us as the neotype: Indemares-Alborán Campaign Sample TE3-DR23 (Fig. 2), collected by Juan Junoy on 25 September 2011 at the depth of 25 m; 35°55.93'N, 03°02.42'W. This specimen is preserved in Bouin’s and stored in 100% ethanol at the Museo Nacional de Ciencias Naturales (MNCN) in Madrid, Spain (Catalog number MNCN 5.02/28). The anterior portion of another individual (TE4-DR23) collected at the same station is stored in 95% ethanol at the Harvard Museum of Comparative Zoology (IZ-132747), and sequences have been published by Kvist et al (2014). See Table 1 for accession numbers, and Table 2 for collecting information.

##### Diagnosis.

Uniformly dark pink (wine) color of the body of living specimens distinguishes *T.antonina* from most species of the genus except *T.hermaphroditica*, *T.arabica* sp. nov. and *T.majinbuui* sp. nov. *Tetranemertesantonina*, *T.majinbuui* sp. nov., *T.arabica* sp. nov., and *T.hermaphroditica* are widely separated geographically, and *T.antonina*, *T.arabica* sp. nov., and *T.majinbuui* sp. nov. are also easily differentiated by DNA barcodes (Table 5). *Tetranemerteshermaphroditica* is a simultaneous hermaphrodite, whereas *T.antonina* we presume to have separate sexes.

##### Habitat.

Quatrefages (1846) reported the species between the shells of vermetids, which form a sort of belt around the rocks (presumably, intertidally) along the coast of Sicily. Bürger (1895) reported it as “infrequent” from subtidal depths of 70–100 m at shoals in the Gulf of Naples (Secca di Benda Palumma, Secca di Chiaia), and at the Blue Grotto of Capri. Specimens used in this study were collected from coralline red algae and *Ulva* at 25 m depth in the western Mediterranean Sea at Alborán Island, Spain.

##### Geographic distribution.

Mediterranean Sea (Banyuls, Trieste, Sicily, Gulf of Naples, Alborán Island, Almería, Strait of Gibraltar).

##### Etymology.

Unknown, but appears to be a derivative of a person’s name.

##### Notes.

The original description (Quatrefages 1846) is very brief. While Quatrefages (1846) did not observe the stylets, noting that the proboscis is unarmed (“proboscis inerme”), Bürger’s much more detailed redescription (1895) includes an illustration of the stylet region of the proboscis, depicting a cylindrical basis that is rounded posteriorly (Fig. 2B). It is not known whether there is variation in the shape of the basis between individuals of this species to range from rounded to forked, as we have observed among specimens of most of the newly described species. Quatrefages may have missed the stylets because *Tetranemertes* species have an unusually short anterior proboscis, with stylets often found within millimeters of cerebral ganglia. This proximity of proboscis armature to the brain often makes it difficult to obtain stylet preparations. Stylets of the recently collected specimens have not been examined. Photographs of recently collected live specimens identified as *T.antonina* and their occurrence records can be found in Herrera-Bachiller et al. (2015) and Herrera-Bachiller (2016). Unfortunately, stylet armature was not observed in the recently collected specimens.

#### ﻿ Tetranemertes rubrolineata

Taxon classificationAnimaliaMonostiliferaOerstediidae

(Kirsteuer, 1965)

B65DF9AE-2EF4-5824-9968-DBFF05D7DCF4

Fig. 3

Nemertes rubrolineata Kirsteuer, 1965: 316; Gibson 1982: 277.

##### Material examined.

The holotype (in the form of histological sections on slides) deposited at the American Museum of Natural History in New York, USA (AMNH 276) was not examined, as external features and stylet characteristics are not discernible in this material. It is not possible to extract DNA from this material. SAM examined several live specimens from the Arabian Sea (Mirbat, Dhofar Governorate, Oman), which conform to the description of *T.rubrolineata*. See Table 1 for specimen details and accession numbers, and Table 2 for collecting information.

##### Diagnosis.

Body color of living specimens (white or yellowish, with a single, longitudinal, wine-red, dorsal stripe) distinguishes *T.rubrolineata* from all other described species of the genus except *T.unistriata* sp. nov. (a look-alike described below). Basis of central stylet thick and bilobed posteriorly (Kirsteuer 1965: fig. 17), twice as long as stylet. DNA sequence data (or tissue for molecular analysis) are not available for *T.rubrolineata* from the type locality (Madagascar) but are available for material from Oman that we consider potentially conspecific.

##### Habitat.

At the type locality (Madagascar) relatively common on *Acroporacytherea* (Dana, 1846) (syn. *Acroporacorymbosa*), *Acroporapharaonis* (Milne Edwards, 1860), *Seriatoporahystrix* Dana, 1846 (syn. *Seriatoporaangulata*), and *Poritesnigrescens* Dana, 1846. In southern Oman (Mirbat) among coral and shell rubble at 8–30 m depth.

##### Geographic distribution.

Madagascar (Tanikely Island, Mozambique Channel), and Arabian Sea (southern Oman).

##### Etymology.

The species epithet refers to the color pattern of living specimens, specifically the red, mid-dorsal, longitudinal stripe.

##### Notes.

**Figure 3.**
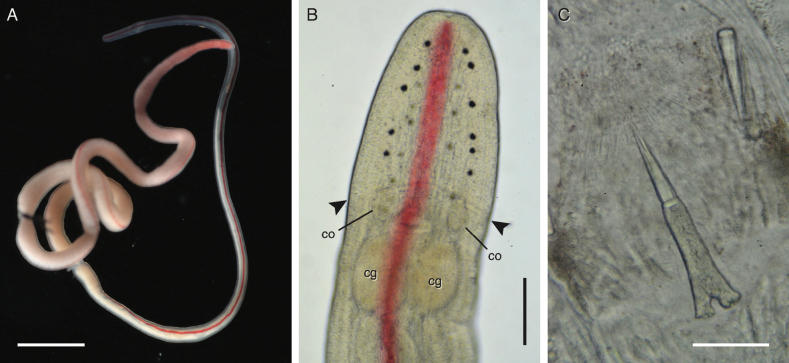
*Tetranemertesrubrolineata* from the Arabian Sea (Oman). Individual BOMAN-8038 **A** external appearance in life **B** head in transmitted light, showing number and distribution of ocelli, as well as ventral cephalic furrow (arrowheads) **C** stylet armature. Abbreviations: cg — cerebral ganglion, co — cerebral organ. Scale bars: 4 mm (**A**); 0.25 mm (**B**); 50 μm (**C**).

SAM collected several specimens resembling *T.rubrolineata* in southern Oman (vicinity of Mirbat) in January 2022 (Fig. 3). These individuals had stylets with helical sculpting and a posteriorly bilobed to deeply forked basis of central stylet (Fig. 3C) in larger individuals (3–4 cm long). The original description of *T.rubrolineata* depicts a slightly bilobed basis, but does not mention sculpted stylets (perhaps the likely quality of the compound microscope in the primitive field conditions of Tanikely Island in 1959 would have made that observation difficult). Two smaller individuals (1–2 cm long) with an undersized proboscis and armature had a posteriorly rounded cylindrical basis, similar to that observed in the small individual of *T.unistriata* sp. nov. from Oman.

#### ﻿ Tetranemertes hermaphroditica

Taxon classificationAnimaliaMonostiliferaOerstediidae

(Gibson, 1982)

461A35FA-E279-5682-89DD-F99609626389

Nemertes hermaphroditicus Gibson, 1982: 227.

##### Type material.

No specimens of this species were available to us for morphological examination or DNA barcoding. Type material consists of the holotype: mature individual, full series of transverse and oblique sections, deposited at the Australian Museum, in Sydney, Australia (W.5880), collected by R. Gibson, 13 July 1975, western mid-reef flat, Heron Island, Capricorn Group.

##### Diagnosis.

Body color of living specimens dusky pink overall with translucent and colorless body margins, resembling *T.antonina*, *T.arabica* sp. nov., and *T.majinbuui* sp. nov. *Tetranemerteshermaphroditica* is the only species in the genus known to be a simultaneous hermaphrodite. Basis of central stylet is described to be cylindrical and rounded posteriorly, similar to that reported by Bürger (1895) for *T.antonina*, and unlike the posteriorly bilobed or deeply forked basis in other species of the genus, at least in larger individuals. Wide geographic separation also supports the distinctiveness of this species from all others.

##### Habitat.

Beneath a large fragment of dead coral (*Acropora* sp.) partially embedded in clean coral sand.

##### Geographic distribution.

Heron Island, Australia.

##### Etymology.

The specific name refers to the fact that specimens of this species are hermaphrodites. Note that both *Nemertes* and *Tetranemertes* are of female gender (Nemertes is a Greek nymph, daughter of the god Nereus). Consequently, the correct Latin ending of the specific epithet should be -a, not -us.

#### ﻿ Tetranemertes bifrost sp. nov.

Taxon classificationAnimaliaMonostiliferaOerstediidae

66ACAAE4-A82B-5D1D-95F5-5BE80C36B517

https://zoobank.org/BD99B99B-0F94-403B-86E2-068BD6993D15

Fig. 4

##### Material examined.

Type material in the form of histological sections, anterior end preserved for histology, and tissue in 95% ethanol is deposited with the Smithsonian Institution’s National Museum of Natural History: holotype CB057_18_07 (USNM 1618678), paratype 852_080203_8 (USNM 1156475), paratype CB057_18_01 (USNM 1618745). See Table 1 for additional specimens and accession numbers, and Table 2 for collecting information.

##### Diagnosis.

*Tetranemertesbifrost* sp. nov. differs from all other species of this genus by its distinctive color: purple to black dorsally, with a pattern of bright iridescent longitudinal stripes and spots, which may appear blue, green, yellow and orange, depending on the type of lighting, background, and individual, and blue ventrally. Additionally, it differs from *T.antonina*, *T.hermaphroditica*, and *T.paulayi* sp. nov. by basis of central stylet posteriorly forked, and from the first two species by having stylets sculpted with spiral groves. At the molecular level, COI barcodes of sequenced specimens clearly differentiate it from other species of *Tetranemertes* (Table 5).

##### Description.

**Figure 4.**
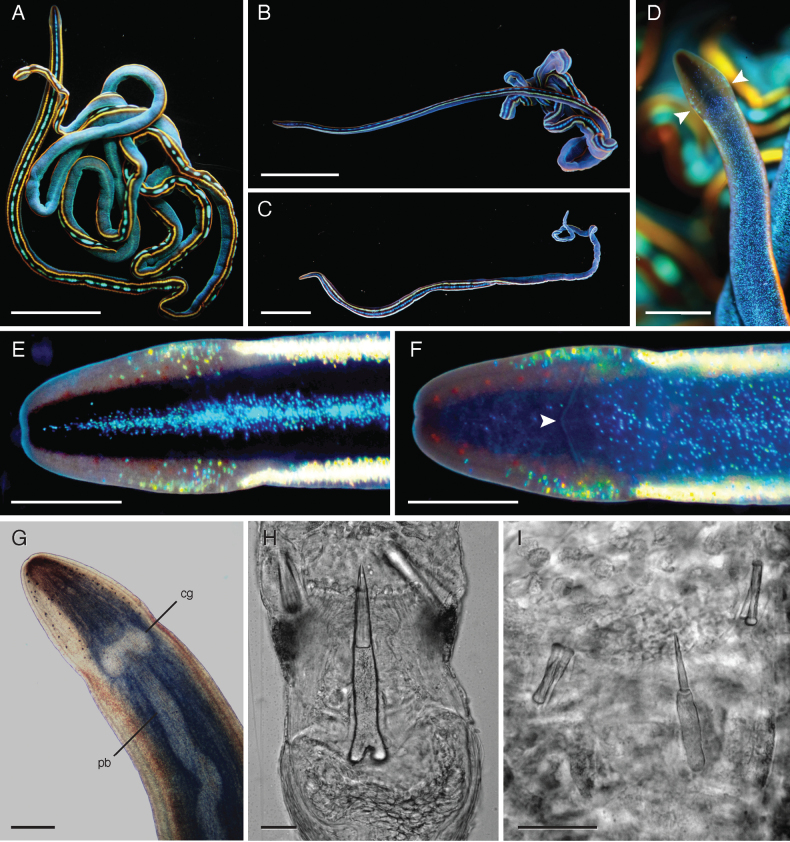
*Tetranemertesbifrost* sp. nov. **A–C** external appearance in life of individuals CBdT0062 (**A**), CB057_18_01 (**B**), and CB057_18_07 (**C**) showing variation in color **D** ventral view of anterior end of individual SMCP0366 showing cephalic furrow (arrowhead) **E, F** close up views of head in dorsal (**E**) and ventral (**F**) view of individual CB057_18_01 showing distribution of pigment, eyes, and the cephalic furrow (arrowhead on **F**) **G** anterior end of individual CB057_18_03 compressed under a glass slide to show number and distribution of eyes **H, I** stylets of individuals CB057_18_01 (**H**) and CB057_18_04 (I). Note the sculpted stylets, and the difference in basis shape (forked or rounded posteriorly). Abbreviations: cg — cerebral ganglion, pb — proboscis. Scale bars: 5 mm (**A–C**); 1 mm (**D**); 0.5 mm (**E–G**); 25 μm (**H, I**).

***External appearance of live specimens*.** Long, thin, thread-like body. Can stretch much more than 200 mm long, and up to 0.5 mm wide at the head; body up to 0.7 mm wide. Body slightly compressed dorso-laterally in cross-section throughout most of its length. The caudal end is rounded with no obvious modifications. *Tetranemertesbifrost* sp. nov. is, perhaps, the most spectacularly colored nemertean in the Caribbean, if not the world (Fig. 4A–F). Larger individuals present a black to deep purple body dorsally, with a discontinuous bright blue mid-dorsal longitudinal stripe running from anterior to posterior tip of body. The blue stripe is flanked by two continuous dorso-lateral stripes that tend to appear orange to yellow in larger individuals, or sky blue to luminescent green in smaller individuals. The flanking stripes begin at the level of the cerebral organ furrow, and continue to the posterior tip of body. The pigment composing the stripes is highly iridescent, so that perceived coloration changes with type and direction of incident light. Ventral side is typically bright blue, pigment scattered over the dark background in a form of numerous tiny speckles (Fig. 4A, D, F). When placed in a dish, worms tangle into a writhing mass, and secrete transparent sticky mucus when handled. Move by ciliary gliding, often with a peristaltic wave passing along the anterior part of the body. When mechanically perturbed, contract into a loose coil. The anterior head region is less contractile, causing an obvious discontinuity in width. Blood is colorless. Remarkably resistant to breaking while being extracted from complex substratum.

The head has a characteristic, narrow diamond or spearhead shape, vaguely reminiscent of a viper’s head, its anterior region demarcated from the rest of body both by width and a single ventral cephalic furrow, formed by the midventral fusion of the cerebral organ furrows. The cephalic furrow is located anterior to the cerebral ganglia, runs from the lateral sides toward the ventral midline forming a shallow forward-pointing “V”. Margins of the head are clear, translucent; the anterior-most mid-dorsal part of the head often reddish brown (Fig. 4G). Small yellow-orange iridescent speckles are scattered on the lateral sides of the head at the level of the cephalic furrow (Fig. 4E). Mouth and rhynchopore combine into a single ventral anterior rhynchostomopore. Posterior cephalic furrows lacking.

Numerous small reddish brown ocelli (10–20 on each side of head) are arranged in four longitudinal rows (Fig. 4E–G). The medial rows, which are not visible in ventral view, reach as far back as anterior margin of cerebral ganglia. The lateral rows have fewer eyes, reach slightly past the cephalic furrow and are visible in ventral view (Fig. 4F). Ocelli are somewhat obscured by the dark pigment of the head, and best seen in worms compressed between a slide and a cover-glass (Fig. 4G). When viewed from dorsal side appear to form two rows (because the two rows on each side of head are almost directly on top of each other).

Cerebral organs are small and inconspicuous, difficult to see in living specimens, even when compressed under a glass slide and viewed under a compound microscope. Anterior to the cephalic furrow, the head is dorso-ventrally flattened; posterior to it, there is a dorsal bulge over the brain, and the body is rounder in cross-section than the anterior tip. The anterior margin of the head is bluntly pointed, indented anteriorly by the rhynchostomopore. The cerebral ganglia are clear, and are located at the base of the “spearhead”, connected by fairly wide cerebral commissures (Fig. 4G).

***Rhynchocoel and proboscis*.** Rhynchocoel does not exceed 1/3 of body length. Proboscis transparent, with two distinctive regions separated by a stylet region. The anterior proboscis is much shorter than the posterior, and the stylet bulb has a length to width ratio of ~ 1. The proboscis is armed with a single central stylet and a pair of lateral pouches holding two accessory stylets each. The shaft of the stylet is straight, sculpted with longitudinal grooves, twisted into a slight spiral (Fig. 4H, I). The basis is rod-shaped with an approximate 1:4 ratio of width to length, and is forked posteriorly (Fig. 4H). The smallest individual in which stylets have been examined had a smaller posteriorly rounded basis and a smaller stylet (Fig. 4I). Stylet to basis length ratio is 1.2–1.3 (*n* = 4). The wall of the rhynchocoel is tinged blue.

***Reproduction*.** Oocytes, measuring 50–80 micron in diameter were noted in specimen CB057_18_03 collected in August 2018.

##### Habitat.

Free living, benthic marine worms inhabiting coral rubble, gravel, and shell hash. Often found stretched between nooks and crannies of the substratum.

##### Geographic distribution.

Caribbean Sea: Bocas del Toro, Panamá, also common at Fuerte Sherman, Colon, Panamá; photographed off Puerto Rico, draped over an unknown fan coral (in 1970s by Smithsonian photographer Kjell Sandved).

##### Etymology.

The name refers to the bright, colorful iridescent stripes and spots characterizing this species. Bifrost, the rainbow bridge in the Norse mythology, reaches between Midgard, the human Earth, and Asgard, the realm of the gods. Some authors state that the name Bifrost means “shimmering path” or “the swaying road to heaven”, and that it might be inspired by the Milky Way.

#### ﻿ Tetranemertes majinbuui sp. nov.

Taxon classificationAnimaliaMonostiliferaOerstediidae

3EDBA29A-B44F-5837-ACE5-8D4357943F63

https://zoobank.org/61B34C97-1A0E-436F-BA88-524852DC7F16

Fig. 5

##### Material examined.

Type material is deposited with the Smithsonian Institution’s National Museum of Natural History. Holotype CB056_18_01 (USNM 1618677): anterior end preserved for histology, and posterior in 95% ethanol. Paratype 856_080303_3 (USNM 1156478): histological sections of anterior end. See Table 1 for additional specimens, accession numbers, and Table 2 for collecting information.

##### Diagnosis.

Pink body color distinguishes *T.majinbuui* sp. nov. from *T.bifrost* sp. nov., *T.rubrolineata*, *T.unistriata* sp. nov., *T.ocelata* sp. nov., *T.pastafariensis* sp. nov., and *T.paulayi* sp. nov. It differs from *T.antonina* and *T.hermaphroditica* by having a posteriorly bilobed basis, and spirally sculpted shaft of stylet. It most resembles *T.arabica* sp. nov. from which it is widely separated geographically. It is currently unknown whether it has separate sexes; if so, then it would distinguish it from *T.hermaphroditica*, which is a simultaneous hermaphrodite. DNA barcodes characterize this species unambiguously, clearly differentiating it from all other sequenced species of the genus (Table 5).

##### Description.

**Figure 5.**
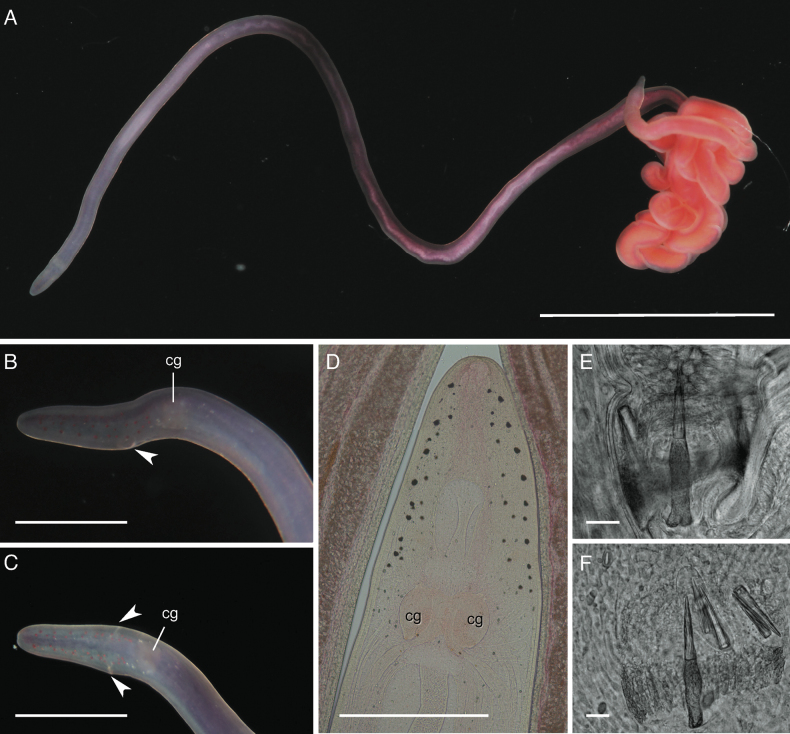
*Tetranemertesmajinbuui* sp. nov. **A** external appearance in life of individual CB056_18_01 **B–D** Close ups of head of a living individual CBdT0058, showing eyes and cephalic furrow (arrowheads) in lateral (**B**) and dorsal (**C**) view. Note the cephalic furrow is on the ventral side, but shows through due to transparency of the body at the anterior end **D** Head compressed under a coverglass to show number and distribution of eyes **E** stylet armature of individual CB056_18_01 with a typical posteriorly bilobed basis **F** stylet armature of individual CBdT0058 with a less typical basis (not bilobed or forked posteriorly). Note helical sculpting of stylet shaft. Abbreviations: cg — cerebral ganglion, co — cerebral organ. Scale bars: 5 mm (**A**); 1 mm (**B–D**); 25 μm (**E, F**).

***External appearance of live specimens*.** Long, thin, thread-like body. Can stretch much more than 10 cm, and is 0.3–0.5 mm wide at the head. Body rounded in cross-section throughout most of its length, but dorso-ventrally compressed in the head region anterior to the brain. The caudal end is bluntly rounded. Body color is uniform intense pink, paler towards the anterior end (Fig. 5A). As viewed with a microscope most of the pink color is associated with the gut, while the anterior end is pale pink or translucent. The caudal end is paler, and has some dorsal granules of darker color. The anterior part of the head is demarcated from the body by both width and a single transverse ventral furrow (Fig. 5B, C, arrowheads).

When placed in a dish, tangles into a writhing mass. Secretes transparent, sticky mucus when handled. Moves by ciliary gliding, often with the anterior end of the head raised at an angle from the surface, the inflection point coinciding with the lateral indentation of the transverse cephalic furrow. When mechanically perturbed, contracts forming a loose coil. The head region anterior to the cephalic furrow is less contractile, causing an obvious discontinuity in width when the animal is contracted. Regeneration ability not known. Blood is colorless.

Head is shaped like a narrow diamond or spearhead, vaguely reminiscent of a viper’s head. Anterior to the cephalic furrow, the head is dorso-ventrally compressed; posterior to it there is a dorsal bulge corresponding to the cerebral ganglia. The cerebral ganglia are clearly visible, transparent or very pale pink.

A single transverse cephalic furrow is formed by a pair of the cerebral organ furrows, fused mid-ventrally. The furrow starts laterally and continues toward the ventral midline, forming a very shallow forward-pointing “V”, anterior to cerebral ganglia (Fig. 5B, C). Rhynchostomopore is ventral. Posterior cephalic furrows lacking.

Numerous reddish brown ocelli (~ 24 on each side of head) are arranged in four longitudinal rows (Fig. 5B–D). The medial rows, which are not visible in ventral view, reach anterior margin of the cerebral ganglia. The lateral rows have fewer eyes, reach slightly posterior to the cephalic furrow and are visible in ventral view. When viewed from dorsal side appear to form two rows, because the two rows on each side of head are almost directly on top of each other. Cerebral organs small and inconspicuous, indiscernible even on squeeze preparations.

***Rhynchocoel and proboscis*.** Rhynchocoel does not exceed 1/3 of body length. The proboscis is translucent, with two distinctive regions separated by a stylet bulb. The anterior region is much shorter than the posterior, and the bulb has a length to width ratio of ~ 1. The proboscis is armed with a single central stylet and a pair of lateral pouches holding two accessory stylets each. The shaft of the stylet is straight, sculpted with spiral grooves (Fig. 5E, F). The basis is rod-shaped with an approximate ratio of 1:4 width to length, characteristically bilobed posteriorly in larger individuals (Fig. 5E). Two individuals with much smaller stylets (possibly, due to younger age or regeneration of proboscis) were observed to have a basis posteriorly rounded rather than bilobed (Fig. 5F). Stylet to basis length ratio is ~ 1:1 (*n* = 1).

***Reproduction*.** No data.

##### Habitat.

Free living, benthic marine worms inhabiting coral rubble at shallow depths (1–7 m). Can be found stretching between nooks and crannies of the substratum.

##### Geographic distribution.

Bocas del Toro, Panamá; Puerto Rico, USA.

##### Etymology.

The species is named after Majin Buu, a male (as far as we can tell) character in the anime Dragon Ball Z, due to its resemblance in color, presence of black dots at the anterior end, and behavior-dependent variability in body width.

#### ﻿ Tetranemertes pastafariensis sp. nov.

Taxon classificationAnimaliaMonostiliferaOerstediidae

9AB8EB2C-6870-520A-9D48-92D62F3DD9FC

https://zoobank.org/6AB9E252-93B8-4EB7-8C8B-72E5902D3A9E

Fig. 6

##### Material examined.

Type material is deposited with the Smithsonian Institution’s National Museum of Natural History. Anterior ends are preserved for histology and posterior — in 95% ethanol: holotype CB055_18_04 (USNM 1618675), paratype CB055_18_06 (USNM 1618676). See Table 1 for additional specimens, accession numbers, and Table 2 for collecting information.

##### Diagnosis.

*Tetranemertespastafariensis* sp. nov. differs from all other described species of this genus, except *T.ocelata* sp. nov. by uniformly pale yellow to orangish yellow body color. It differs from *T.ocelata* sp. nov. by having smaller ocelli. Easily differentiated from other species with DNA sequence data (Table 5).

##### Description.

**Figure 6.**
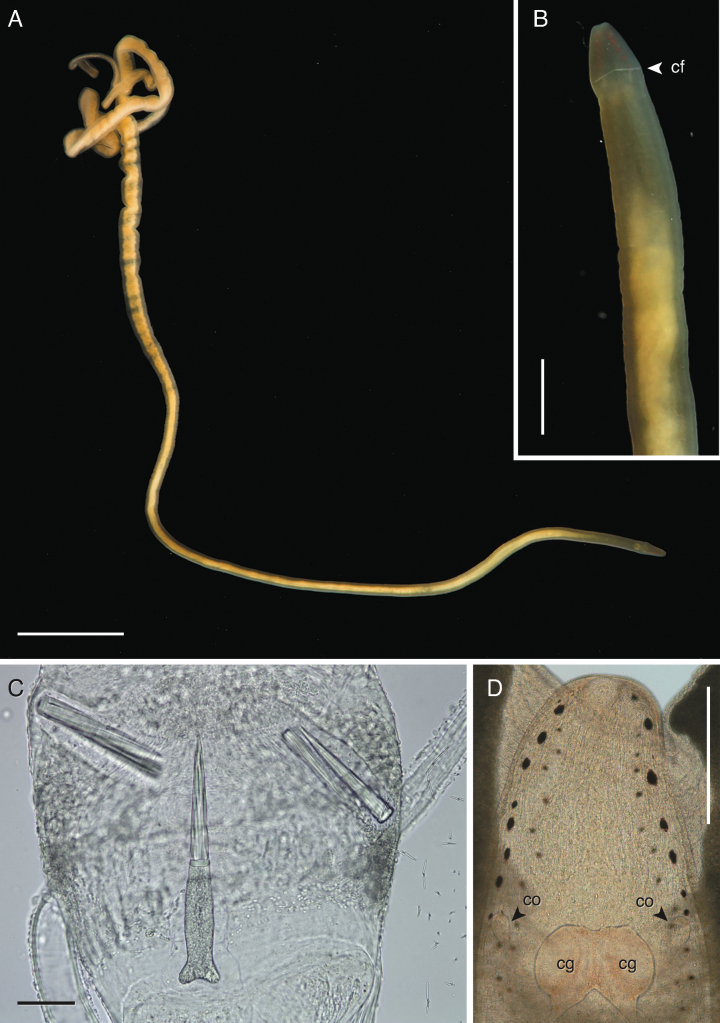
*Tetranemertespastafariensis* sp. nov. **A** external appearance in life of individual CB055_18_04 **B** anterior end in ventral view of individual CB055_18_06 showing cephalic furrow (arrowhead, cf) **C** stylets of individual CB055_18_08 showing a posteriorly bilobed basis and sculpted stylets **D** head of individual CBdT0059 compressed under a glass slide showing number and distribution of eyes, as well as cerebral organs (arrowheads) and pinkish cerebral ganglia. Abbreviations: cg — cerebral ganglion, co — cerebral organ. Scale bars: 5 mm (**A**); 1 mm (**B, D**); 25 μm (**C**).

***External appearance of live specimens*.** Long, thin, thread-like body can stretch more than 10 cm in length and is up to 0.5 mm wide at the head; body up to 1 mm wide. Body rounded or slightly compressed in cross-section. The caudal end is rounded. Body color is uniform orangish yellow, paler towards the anterior end (Fig. 6A, B). Blood is colorless.

When placed in a dish, often forms a writhing tangle. When mechanically disturbed it contracts into a knot, and secretes transparent sticky mucus. Moves by ciliary gliding, often with the anterior end of the head raised from the surface, the inflection point coinciding with the lateral indentation of the anterior cephalic furrow. The head region anterior to the anterior furrow is less contractile, causing an obvious discontinuity in width when the anterior end of the animal is contracted. Regeneration ability not known.

The head is somewhat triangular, spear-shaped, resembling head of a snake. The anterior part of the head is demarcated from the body by both width and an anterior ventrolateral cephalic furrow. The body is dorso-ventrally flattened in front of the anterior cephalic furrow; and thicker posterior to it, with a dorsal bulge corresponding to the brain. The cerebral ganglia, tinged pale pinkish orange, are visible through the body wall. The anterior margin of the head is bluntly pointed, slightly indented by the rhynchostomopore.

Cerebral organ furrows are fused ventrally forming a single cephalic furrow (Fig. 6B, arrowhead). The cephalic furrow wraps around the lateral sides of head, and is barely visible from the dorsal side as a pair of lateral indentations. On the ventral side it forms a shallow anteriorly-pointed “V” anterior to cerebral ganglia. Posterior cephalic furrow is lacking. Mouth and rhynchopore are combined into a single anterior ventral rhynchostomopore.

Ocelli (20–40) are arranged in four longitudinal rows, two on each side of the head (Fig. 6B), reaching to posterior margin of cerebral ganglia (Fig. 6D), black in transmitted light. The medial rows are not visible from the ventral side. Cerebral organs present, clearly visible in specimens compressed under a cover slide (Fig. 6D).

***Rhynchocoel and proboscis*.** Rhynchocoel does not exceed 1/3 of body length. The proboscis is transparent, with two distinctive regions separated by a proboscis bulb. The anterior region is much shorter than the posterior, and the bulb has a length to width ratio of ~ 1. Proboscis armed with a single central stylet and two pouches, each holding two or three accessory stylets. The central stylet shaft is straight with spiral grooves. The stylet basis is rod-shaped, with a width to length ratio of ~ 1:4 to 1:5, with characteristic shallow fork posteriorly (Fig. 6C). One individual had a cylindrical basis with rounded posterior (not shown). Stylet to basis length ratio is ~ 1:1 (*n* = 2).

***Reproduction*.** No data.

##### Habitat.

Free living, marine benthic species, inhabiting shallow coral reef rubble (1–7 m depth). Tends to stretch in the crannies of the substratum.

##### Geographic distribution.

Currently only known from Bocas del Toro, Panamá.

##### Etymology.

The species is named after its resemblance to the Flying Spaghetti Monster, the deity of the Pastafarian religion.

#### ﻿ Tetranemertes unistriata sp. nov.

Taxon classificationAnimaliaMonostiliferaOerstediidae

36F9C13F-DA26-53B1-8A8D-64B87D8AD7A0

https://zoobank.org/9FB78688-612F-4270-A2E7-7EC25451D2BD

Fig. 7

##### Diagnosis.

*Tetranemertesunistriata* sp. nov. differs from most other described species of the genus by its color pattern: a single pale to dark pink mid-dorsal longitudinal stripe on a pale yellow to pale pinkish background. Resembles *T.rubrolineata* from Madagascar (Kirsteuer 1965) in body color. Although tissue for DNA analysis is not available for *T.rubrolineata* from Madagascar, DNA sequences from individuals collected by SAM from the Arabian Sea and identified as *T.rubrolineata* clearly separate the two species. DNA barcodes of *T.unistriata* sp. nov. are also clearly distinct from those of all other sequenced species of the genus (Table 5).

##### Material examined.

Type material in the form of histological sections and tissue in 95% ethanol is deposited with the Smithsonian Institution’s National Museum of Natural History. Holotype: 490_071099_2 (USNM 1011574), paratype 490_070999_1 (USNM 1011573). Additional specimens, accession numbers, and collecting information can be found in Tables 1, 2.

##### Description.

**Figure 7.**
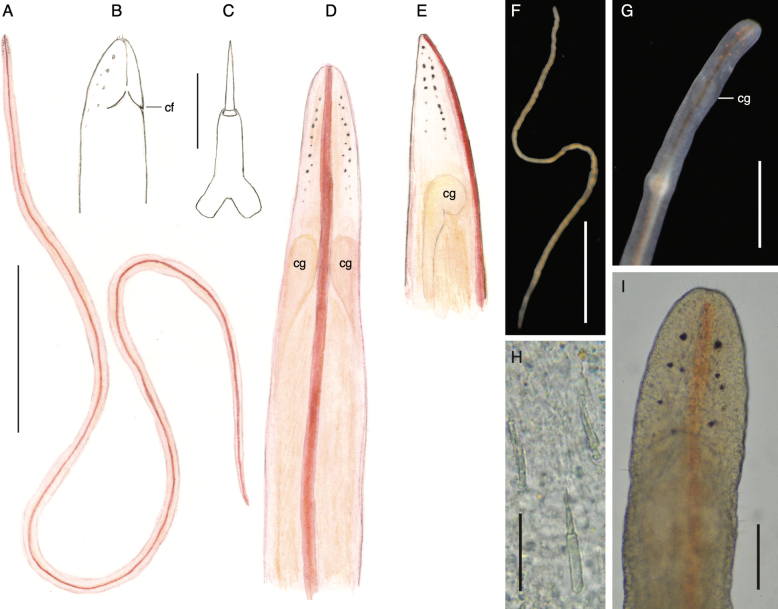
*Tetranemertesunistriata* sp. nov. **A–E** based on sketches from live material (individuals 490_070999_1 through 490_070999_3) from Japan **A** external appearance in life **B** diagram of head in ventro-lateral view showing cephalic furrow **C** stylet and basis **D, E** anterior end in dorsal (**D**) and lateral (**E**) views showing number and arrangement of eyes, and cerebral ganglia **E** stylet and basis **F, G** appearance in life of individual BOMAN-07032 from Oman: body shape (**F**), anterior end in incident light (**G**) showing size and position of cerebral ganglia and eyes, stylet apparatus (**H**), and compressed head region in transmitted light to show eye arrangement (I). Abbreviations: cf — cephalic furrow, cg — cerebral ganglion. Scale bars: 1 cm (**A**); 50 μm (**C**); 5 mm (**F**); 1 mm (**G**); 40 μm (**H**); 150 μm (**I**).

***External appearance of live specimens*.** Body thin, thread-like, a few centimeters in length, and less than a millimeter in width. Anterior and posterior ends are gently tapering, bluntly rounded. Color in life varies from pale yellowish orange to pale pink, with a continuous thin reddish or dark pink mid-dorsal longitudinal stripe that reaches from anterior to posterior tip of body (Fig. 7A). Head the same width or slightly narrower than the rest of body, demarcated from the rest of body by a pair of ventral anterior cephalic furrows, nearly fused mid-ventrally to form a shallow anteriorly pointed “V” (Fig. 7B). Rhynchostomopore appears as a short ventral slit, at the anterior tip of head. Small dark ocelli (9–12 on each side of head) are arranged in four longitudinal rows, the two rows on each side of head almost directly on top of one another (Fig. 7D, E). The smaller individual from Oman had fewer ocelli (Fig. 7G, I). Cerebral ganglia large, yellowish or pinkish, partly translucent, show through the body wall (Fig. 7D, E, G). Cerebral organs small, inconspicuous.

***Rhynchocoel and proboscis*.** Rhynchocoel length unknown, but likely restricted to anterior-most region of body. Proboscis short and thin. Stylets were examined in one ~ 8-cm long individual (paratype) from Japan, and the ~ 2 cm long individual from Oman. Central stylet with a straight spirally sculpted shaft, 20–50 μm long. Basis cylindrical, deeply forked posteriorly, 25–75 μm long (Fig. 7E). Two accessory stylet pouches with 2 stylets each. Smaller individual from Oman had considerably smaller stylets and smaller cylindrical basis, rounded posteriorly (Fig. 7H).

***Reproduction*.** No data.

##### Habitat.

Free-living, marine. Among brown and branched calcareous algae at the depths of 1–2 m in the type locality in Japan. Among coral rubble at 4–9 m depths in Oman.

##### Geographic distribution.

Type locality is near Seto Marine Laboratory on the Pacific coast of Honshu Island, Japan. One other individual was collected by SAM in the Arabian Sea (Dhofar Governorate, Oman).

##### Etymology.

Specific epithet reflects the color pattern of living individuals.

#### ﻿ Tetranemertes ocelata sp. nov.

Taxon classificationAnimaliaMonostiliferaOerstediidae

2B679194-9313-52DE-AE78-37A5204F5EF8

https://zoobank.org/0B252009-5B4D-46FB-8105-14C2D03AF251

Fig. 8

##### Diagnosis.

*Tetranemertesocelata* sp. nov. differs from most other species of the genus by uniformly pinkish orange body color without distinct markings. It most resembles *T.pastafariensis* sp. nov., from which it differs by having much larger ocelli, and more intense color (pinkish orange as opposed to pale yellow). It differs from *T.paulayi* sp. nov. by having larger eyes, paler body color, and colorless blood. DNA sequences also clearly differentiate this species from all other sequenced species of the genus (Table 5).

##### Material examined.

Type material in the form of histological sections is deposited with the Smithsonian Institution’s National Museum of Natural History: holotype 685_061202_2 (USNM1156296), paratype 685_061202_4 (USNM 1156298). See Table 1 for additional specimens, accession numbers, and Table 2 for collecting information.

##### Description.

**Figure 8.**
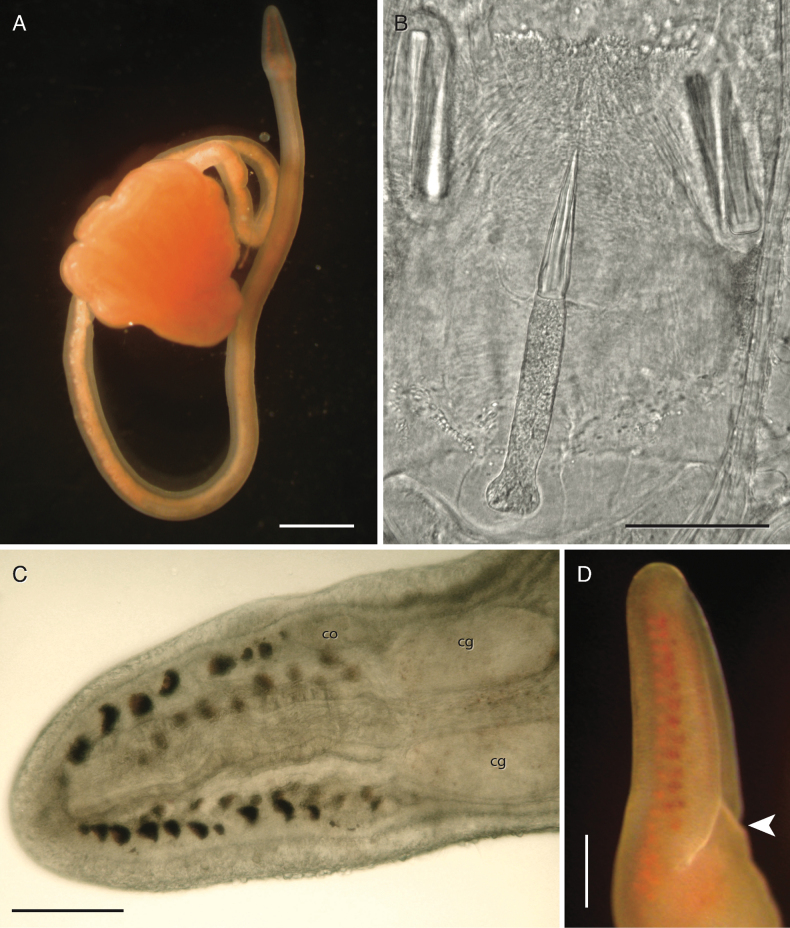
*Tetranemertesocelata* sp. nov. **A** external appearance in life **B** proboscis armature showing bilobed basis and sculpted stylets **C** head compressed under coverslip to show number and arrangement of eyes, cerebral organs and cerebral ganglia **D** head in ventro-lateral view showing eyes and the cephalic furrow (arrowhead). Abbreviations: cg — cerebral ganglion, co — cerebral organ. Scale bars: 1 mm (**A**); 50 μm (**B**); 0.2 mm (**C, D**).

***External appearance of live specimens*.** Body long and thin, thread-like, tangles easily, up to 12 cm long at rest, but can stretch up to 50 cm. Body width varies from 0.1 mm posteriorly to 0.7 mm in the head region. Head dorso-ventrally flattened; the rest of the body cylindrical in cross-section. Most of the time the worm remains loosely and irregularly tangled and coiled. The head contracts linearly when disturbed. Background color from very pale yellow to salmon color or deep pinkish orange (Fig. 8A). The epidermis appears pale and translucent to transparent, the deepest color associated with the gut. Small specks of darker orange or pale brown pigment scattered throughout the epidermis, others associated with the central nervous system.

Head slightly wider than adjacent body, triangular or spear-shaped, reminiscent of the shape of a snake’s head. Anterior tip of head bluntly rounded. Head demarcated from the body by a single shallow ventro-lateral cephalic furrow, formed by a pair of cerebral organ furrows meeting mid-ventrally, and creating an anteriorly directed “V” (Fig. 8D). Posterior cephalic furrow is lacking. Mouth and rhynchopore are combined into a single anterior ventral rhynchostomopore. The head is widest at the level of the cephalic furrow.

Ocelli are proportionally much larger than in other species of the genus, resemble those of cratenemertids and reptant polystiliferans, reddish brown in color, arranged in four rows (two on each side of head, almost directly on top of each other). Eyes of the more lateral/ventral rows are larger than the eyes in the medial/dorsal rows (Fig. 8C, D).

Cerebral ganglia large, translucent, with specks of pinkish pigment, and very wide commissures. Cerebral organs anterior to the cerebral ganglia, visible on squeeze preparations of the head (Fig. 8C).

***Rhynchocoel and proboscis*.** Length of rhynchocoel unknown. Proboscis very short (a few mm long), restricted to the anterior-most part of body. Basis of central stylet long and cylindrical, variably rounded or slightly bilobed posteriorly . Shaft of central stylet straight, sculpted with weakly spiraling groves. Two accessory stylet pouches, each with two accessory stylets (Fig. 8B).

***Reproduction*.** Reproductive specimens collected at Carrie Bow Cay, Belize in June 2002.

##### Habitat.

Subtidal coral and shell rubble (with significant quantity of orange sponge) at the depth of 20–71 m depth.

##### Geographic distribution.

Caribbean Sea (Belize) and the Gulf of Mexico.

##### Etymology.

Specific epithet refers to the size of the ocelli, which are larger than in the other described species of this genus.

#### ﻿ Tetranemertes paulayi sp. nov.

Taxon classificationAnimaliaMonostiliferaOerstediidae

6D34315B-7E0B-5C92-BBCF-1ADF61E0B7FF

https://zoobank.org/4854CAEC-0C27-467A-8E23-29C15E7FD448

Fig. 9A–C

##### Diagnosis.

*Tetranemertespaulayi* sp. nov. differs from all other known species of the genus by its orange color, and reddish orange blood vessels. Also, all examined specimens of this species, including the largest, had a pear-shaped rounded basis of central stylet, never bilobed or forked, unlike in larger individuals of most other species of the genus that we have examined. DNA barcoding clearly shows that this species is distinct from the other representatives of the genus (Table 5).

##### Material examined.

Type material in the form of anterior and posterior preserved for histology, and midbody in 95% ethanol is deposited with the Florida Museum of Natural History. Holotype: BOMAN_07013 (UFID 1055), paratype BOMAN_08291 (UFID 1118). See Table 1 for additional specimens, accession numbers, and Table 2 for collecting information.

##### Description.

**Figure 9.**
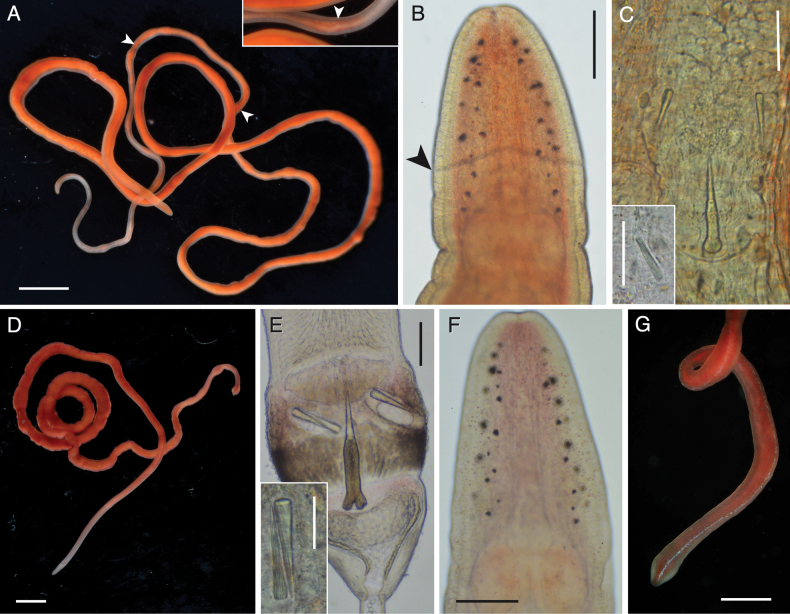
*Tetranemertesarabica* sp. nov. and *Tetranemertespaulayi* sp. nov. from the Arabian Sea (Oman), and *Tetranemertes* sp. ETP001 from Eastern Tropical Pacific **A–C***Tetranemertespaulayi*. Individual BOMAN-07013, holotype, external appearance in life (**A**), and head compressed under coverglass (**B**) to show number and arrangement of eyes, and cephalic furrow (arrowhead). Note orange blood vessels (inset, and white arrowheads). Individual BOMAN-08291, paratype (**C**), stylets, viewed through the body wall of an individual compressed under coverglass (hence the orange tinge), inset emphasizes sculpted stylets (individual BOMAN-08302) **D–F***Tetranemertesarabica*. Individual BOMAN-09099, external appearance in life (**D**), proboscis armature (**E**). Inset on E shows sculpted stylets (individual BOMAN-08030). Individual BOMAN-08050, head compressed under coverslip to show arrangement of eyes (**F**). Note the pink tinge to the body wall and proboscis wall, and orangish cerebral ganglia **G***Tetranemertes* sp. ETP001 from the Pacific coast of Panamá (individual SMPP0632). Scale bars: 3 mm (**A**); 0.2 mm (**B, F**); 50 μm (**C** and insets on **C, E**); 100 μm (**E**); 2 mm (**D**); 0.5 mm (**G**).

***External appearance of live specimens*.** Body is orange-colored (Fig. 9A), with pigment mostly associated with subepidermal layers and the gut. The epidermis pale and translucent, with small specks of orange color throughout. Smaller individuals are paler in color. Body shape is typical for species of the genus: long and thread-like, widest at the level of cerebral ganglia. Anterior tip bluntly rounded. Cerebral organs far in front of the cerebral ganglia (approximately half-way between the cerebral ganglia and anterior tip of head). A single ventro-lateral cephalic furrow is in front of cerebral ganglia, shaped as a shallow anteriorly directed “V” (Fig. 9B). Posterior cephalic furrow is lacking. The head is widest at the level of the cephalic furrow. Blood vessels appear orangish red, and stand out against the background of other internal structures, especially in the posterior region.

***Rhynchocoel and proboscis*.** Rhynchocoel limited to the anterior-most part of the body (~ ¼ of body length). Proboscis is very short, with stylet region found immediately posterior to the cerebral ganglia. Basis of central stylet is consistently rounded, never forked or bilobed, even in the largest individuals (4–6 cm long). The stylet shaft is spirally sculpted (Fig. 9C).

***Reproduction*.** No data.

##### Habitat.

Coral rubble, vermetid-coralline encrustations on rocks, algal holdfasts, barnacles, and algae, at depths of 0–13 m.

##### Geographic distribution.

Currently only known from the Arabian Sea (Mirbat, Dhofar Governorate, Oman).

##### Etymology.

Species is named after Dr. Gustav Paulay for his outstanding contributions to studies of marine invertebrate diversity of the world.

#### ﻿ Tetranemertes arabica sp. nov.

Taxon classificationAnimaliaMonostiliferaOerstediidae

AB297EC8-BF23-5499-942D-7694E92F68D5

https://zoobank.org/582875FE-63F3-4178-A3B3-A123DFA8BD7A

Fig. 9D–F

##### Diagnosis.

Pink body color distinguishes *T.arabica* sp. nov. from *T.bifrost* sp. nov., *T.rubrolineata*, *T.unistriata* sp. nov., *T.ocelata* sp. nov., *T.pastafariensis* sp. nov., and *T.paulayi* sp. nov. It differs from *T.antonina* and *T.hermaphroditica* by having a basis posteriorly bilobed, stylet spirally sculpted. It most resembles *T.majinbuui* sp. nov. from which it is widely separated geographically. It is currently unknown whether it has separate sexes; if so, then it would distinguish it from *T.hermaphroditica*, a simultaneous hermaphrodite. DNA barcodes characterize this species unambiguously, clearly differentiating it from all other sequenced species of the genus (Table 5).

##### Material examined.

Type material is deposited with the Florida Museum of Natural History. Holotype BOMAN_08050 (UFID 1087), in the form of anterior and posterior preserved for histology, and midbody in 95% ethanol, and paratype BOMAN_08300 (UFID 1085) in the form of anterior and posterior preserved for histology, and midbody in 95% ethanol. See Table 1 for additional specimens, accession numbers, and Table 2 for collecting information.

##### Description.

***External appearance of live specimens*.** Body is pale to deep pink. Larger individuals have deeper coloration. Pink specks scattered throughout epidermis, as well in the stylet region of the proboscis. Body long and thin, thread-like, widest at the level of the cerebral ganglia and cephalic furrow (Fig. 9D). Anterior end is least pigmented, semi-translucent. Eyes (25–35) are arranged in four longitudinal rows, two rows on top of each other on either side of head. Anterior tip of head bluntly rounded. A single ventro-lateral cephalic furrow is in front of cerebral ganglia, shaped as a shallow anteriorly directed “V” (Fig. 9F). Posterior cephalic furrow is lacking.

***Rhynchocoel and proboscis*.** Rhynchocoel and proboscis very short (< 1/3 body length), with stylets found within the anterior-most quarter of the body. All examined individuals (*n* = 8) possessed a posteriorly forked basis and spirally sculpted stylets (Fig. 9E).

***Reproduction*.** Ripe males observed in January 2022.

##### Habitat.

Coral rubble, shell hash, rocks encrusted with coralline algae and vermetid tubes; between the depths of 2–30 m.

##### Geographic distribution.

Currently only known from the Arabian Sea (Mirbat, Dhofar Governorate, Oman).

##### Etymology.

Species epithet refers to the type region of the species, the Arabian Sea (and the coast of the Arabian Peninsula).

#### ﻿ Tetranemertes

Taxon classificationAnimaliaMonostiliferaOerstediidae

sp. ETP001

C420A5BE-EE10-5A9A-8FE2-738303C922D8

Fig. 9G

##### Remarks.

A single individual of this species (SMPP0632) was extracted by CE from coral rubble collected by Maycol Madrid on 20 Jan 2020 at Isla Pachequilla (8.67231, -79.0605) from a depth of 5 m using SCUBA. Posterior end of specimen was missing. Partial specimen was 4–5 mm long and ~ 0.3 mm wide. Body uniformly pink with colorless margins and a single mid-dorsal longitudinal stripe, which is light in color and iridescent, similar to that seen in the Caribbean species *Tetranemertesbifrost* sp. nov. described above, and begins just posterior to the cephalic lobe. Four rows of pre-cerebral ocelli, two on each side, orange-red in reflected light, large in relation to head compared to other eumonostiliferan nemerteans. Head wider than body, diamond-shaped. Stylets not observed. Although we do not have a morphological voucher to designate the holotype and thus formally describe this species, DNA sequences clearly differentiate it from all other species of *Tetranemertes* (Table 5). See Table 1 for accession numbers.

## ﻿Discussion

The present study expands the number of known species within the genus *Tetranemertes* from three to 11, including seven newly described and one undescribed species. This is the first report of *Tetranemertes* species in the Caribbean Sea, the Gulf of Mexico, the North Pacific Ocean (Japan and Panamá), and the Arabian Sea (Oman). *Ommatopleaophiocephala* described from South Africa by Schmarda (1859), redescribed by Wheeler (1934), and synonymized with *T.antonina* by Friedrich (1955) on the basis of having anteriorly divided longitudinal musculature of the body wall, is almost certainly a distinct species, and likely not related to *Tetranemertes* based on its overall appearance. Generic placement of this species is currently uncertain pending collection of fresh material suitable for DNA sequencing.

Friedrich (1955), in redefining the genus *Nemertes*, placed a lot of emphasis on the fact that its type species, *T.antonina*, possesses longitudinal body muscles anteriorly divided by a layer of extracellular tissue. *Tetranemertesrubrolineata* and *T.hermaphroditica* also share this character. However, this character is not unique to the genus *Tetranemertes*. Kirsteuer (1974) reviewed monostiliferan genera with anteriorly divided longitudinal muscles, which also include *Paranemertes* (in part), and *Poseidonemertes*. It remains unknown whether this character is shared by the newly described species of *Tetranemertes*. However, regardless of the outcome of any future histological investigations, the phylogenetic evidence unambiguously places the newly described species within *Tetranemertes*, with support from other morphological characters. We hold the opinion that histological investigation is unnecessary for routine species descriptions of most nemerteans, and that it precludes rapid characterization of undescribed diversity.

The monophyly of the genus *Tetranemertes* is strongly supported by the molecular phylogenetic analyses, which include sequences from *T.antonina*, *T.rubrolineata*, the seven newly described species, as well as the undescribed species we report from the Pacific coast of Panamá. *Tetranemerteshermaphroditica* currently lacks DNA sequence data, and is included in the genus on morphological grounds alone. Although the genus lacks unique morphological synapomorphies, all included species are similar to *T.antonina* in body shape, morphology of cephalic furrows, distribution of ocelli, and possessing a relatively short rhynchocoel and proboscis. Furthermore, six of the seven new species described here, as well as *T.rubrolineata*, possess an unusual character of having a central stylet basis that is slightly bilobed to deeply forked posteriorly. Observed variation in the shape of the basis among individuals of *T.bifrost* sp. nov., *T.majinbuui* sp. nov., *T.rubrolineata*, *T.unistriata* sp. nov., and *T.arabica* sp. nov., with some individuals having a forked basis and others (usually with much smaller stylets) a posteriorly rounded basis, may reflect changes during ontogeny or regeneration of proboscis. The only species so far in which all examined individuals had rounded basis of central stylet is the undescribed species from Oman, *T.paulayi* sp. nov., which also has the unusual characteristic of having pigmented blood. A forked basis of central stylet may be a unique morphological synapomorphy of the genus, with *T.paulayi* sp. nov. representing a secondary loss of this feature.

## ﻿Conclusions

The current revision of the genus *Tetranemertes* raises the number of known species from three to eleven, including seven newly described, and one undescribed species. Previously, DNA sequence data existed for only two species of the genus, *T.antonina* and *T.bifrost* sp. nov., the latter previously undescribed. We publish first DNA sequence data for one previously described, seven newly described, and one undescribed species; provide a revised morphological diagnosis of the genus, including a possible morphological synapomorphy of a forked basis of central stylet; and provide the first evidence of monophyly based on a multi-gene molecular phylogeny of the genus. New additions to the genus greatly expand the known geographic range of the genus from the Mediterranean, the Great Barrier Reef of Australia, and Madagascar to the Pacific coast of Japan, the Arabian Sea, Eastern Tropical Pacific, and the Caribbean Sea.

## Supplementary Material

XML Treatment for Tetranemertes

XML Treatment for Tetranemertes antonina

XML Treatment for Tetranemertes rubrolineata

XML Treatment for Tetranemertes hermaphroditica

XML Treatment for Tetranemertes bifrost

XML Treatment for Tetranemertes majinbuui

XML Treatment for Tetranemertes pastafariensis

XML Treatment for Tetranemertes unistriata

XML Treatment for Tetranemertes ocelata

XML Treatment for Tetranemertes paulayi

XML Treatment for Tetranemertes arabica

XML Treatment for Tetranemertes

## ﻿Additional information

### Author ORCIDs

Irina Cherneva https://orcid.org/0000-0002-5533-6527

Christina I. Ellison https://orcid.org/0000-0002-1856-386X

Eduardo E. Zattara https://orcid.org/0000-0002-9947-9036

Jon L. Norenburg https://orcid.org/0000-0001-7776-1527

Megan L. Schwartz https://orcid.org/0000-0001-5318-2838

Juan Junoy https://orcid.org/0000-0002-6125-558X

Svetlana A. Maslakova https://orcid.org/0000-0002-3629-6638

### Data availability

All of the data that support the findings of this study are available in the main text. Specimen data is available in the BOLD dataset https://dx.doi.org/10.5883/DS-TETRA.
